# Long non‐coding RNAs in motor neuron development and disease

**DOI:** 10.1111/jnc.15198

**Published:** 2020-10-10

**Authors:** Vamshidhar R. Vangoor, Andreia Gomes‐Duarte, R. Jeroen Pasterkamp

**Affiliations:** ^1^ Department of Translational Neuroscience University Medical Center Utrecht Brain Center Utrecht University Utrecht The Netherlands

**Keywords:** long non‐coding RNA, circular RNA, motor neuron development, spinal muscular atrophy, amyotrophic lateral sclerosis

## Abstract

Long non‐coding RNAs (lncRNAs) are RNAs that exceed 200 nucleotides in length and that are not translated into proteins. Thousands of lncRNAs have been identified with functions in processes such as transcription and translation regulation, RNA processing, and RNA and protein sponging. LncRNAs show prominent expression in the nervous system and have been implicated in neural development, function and disease. Recent work has begun to report on the expression and roles of lncRNAs in motor neurons (MNs). The cell bodies of MNs are located in cortex, brainstem or spinal cord and their axons project into the brainstem, spinal cord or towards peripheral muscles, thereby controlling important functions such as movement, breathing and swallowing. Degeneration of MNs is a pathological hallmark of diseases such as amyotrophic lateral sclerosis and spinal muscular atrophy. LncRNAs influence several aspects of MN development and disruptions in these lncRNA‐mediated effects are proposed to contribute to the pathogenic mechanisms underlying MN diseases (MNDs). Accumulating evidence suggests that lncRNAs may comprise valuable therapeutic targets for different MNDs. In this review, we discuss the role of lncRNAs (including circular RNAs [circRNAs]) in the development of MNs, discuss how lncRNAs may contribute to MNDs and provide directions for future research.

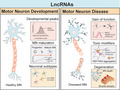

## Graphical abstract

During development, distinct classes of long non‐coding RNAs (lncRNAs) display specific spatiotemporal patterns to drive motor neuron (MN) differentiation and maturation leading to the generation of functional subsets of MNs. MN diseases such as ALS are characterized by changes in the expression of specific classes of lncRNAs and mutant antisense (AS) RNAs, resulting in disease phenotypes. These include the formation of aberrant RNA accumulations (foci) that sequester and interfere with RNA‐binding protein (RBP) function and the production of toxic peptides (DPRs). These changes ultimately lead to MN degeneration and muscle atrophy.

**Abbreviations**
5‐FU: 5‐fluorouracil
AAV: adeno‐associated virus
ABA: Allen brain atlas
AD: Alzheimer's disease
ADARB2: adenosine deaminase RNA‐specific B2 (inactive)
Ago2: Argonaute2
ALS: amyotrophic lateral sclerosis
arcRNAs: architectural RNAs
ASOs: antisense oligonucleotides
*ATXN2*: Ataxin‐2
BDNF: brain‐derived neurotrophic factor
bHLH: basic helix‐loop‐helix
bp: base pairs
C9‐ALS: C9ORF72‐associated ALS
*C9ORF72*: chromosome 9 ORF 72
ChIP: chromatin immunoprecipitation
circRNAs: circular RNAs
CLIP: cross‐linking immunoprecipitation
CNS: central nervous system
CpG: cytosine‐phosphorus‐guanine
DBPs: DNA‐binding proteins
*Dbr1*: debranching enzyme 1
DE: differential expression
DOX: doxorubicin
DPR: dipeptide repeat
dsRNA: double‐stranded RNA
ER: endoplasmic reticulum
ESC: embryonic stem cells
FACS: fluorescence‐activated cell sorting
fALS: familial ALS
FTD: frontotemporal dementia
FUS: fused in sarcoma
FUS/TLS: FUS/translocated in liposarcoma
GFP: green fluorescent protein
HCC: hepatocellular cancer
hESC: human embryonic stem cells
hiPSC: human‐induced pluripotent stem cells
Hox: homeobox
hpf: hours post‐fertilization
*hsrω*: heat‐shock RNA ω
HUVECs: human umbilical vein endothelial cells
IL: interleukins
INF: interferon
KCl: potassium chloride
LMC‐MNs: limb innervating lateral motor column‐motor neurons
lncRNAs: long non‐coding RNAs
LPS: lipopolysacharide
*Malat1*: metastasis‐associated lung adenocarcinoma transcript 1
MATR3: Matrin 3
MEFs: mouse embryonic fibroblasts
*Meg3*: maternally expressed gene 3
mESC: mouse embroynic stem cells
*Miat*: myocardial infarction‐associated transcript
miRNAs: MicroRNAs
MND(s): motor neuron disease(s)
MN(s): motor neuron(s)
MN‐TFs: MN transcription factors
MNX1: Motor neuron and pancreas homeobox 1
NAc: nucleus accumbens
NAT: naturally occurring antisense transcript
NBs: nuclear bodies
ncRNA: non‐coding RNA
*NEAT1*: nuclear‐enriched abundant transcript 1
NONO: non‐PoU domain‐containing octamer‐binding protein
*Olig2*: oligodendrocyte transcription factor 2
PAHs: polycyclic aromatic hydrocarbons
PBLCs: peripheral blood lymphocytes
PBMCs: peripheral blood mononuclear cells
PcG: polycomb group protein
PD: Parkinson's disease
PiRNAs: Piwi‐interacting RNAs
PLDs: prion‐like‐domains
PRC1/2: polycomb repressive complex 1/2
PSPs: paraspeckle proteins
RA: retinoic acid
RAN: repeat‐associated non‐ATG‐initiated translation
RBM14: RNA‐binding motif protein 14
RBPs: RNA‐binding proteins
*Rmst*: rhabdomyosarcoma 2‐associated transcript
RNA‐seq: RNA sequencing
rRNA: ribosomal RNA
sALS: sporadic ALS
*Sat III*: satellite III repeat RNA
SCA2: spinocerebellar ataxia type 2
SCZ: schizophrenia
SFPQ: splicing factor proline/glutamine‐rich
SINEs: short interspersed nuclear elements
SiRNAs: small interfering RNAs
*Slc32a1*: solute carrier family 32 member 1
SMA: spinal muscular atrophy
SMN: spinal motor neuron
SN: substantia nigra
sncRNA: small non‐coding RNA
SO: sense oligonucleotides
*SOD1*: superoxide dismutase1
SSOs: splice‐switching oligonucleotides
TARDBP: TAR DNA‐binding protein
TDP‐43: TAR DNA‐binding protein 43
TLR: toll‐like receptors
UTR: untranslated region
*VAPB*: vesicle‐associated membrane protein‐associated protein B/C
Xist: X‐inactive‐specific transcript

## 1 INTRODUCTION

Over the past decade, the advent of high‐throughput sequencing technologies coupled with advanced bioinformatics analysis has allowed an in‐depth look into the non‐coding part of the genome with exceptional resolution and scale. This has revolutionized our understanding of mammalian genome architecture, activity and regulation and led to the discovery that only ±2% of the human genome encodes proteins. This is remarkable given that the majority of nucleotides are transcribed, thus giving rise to an exquisitely high number of non‐coding RNAs (ncRNAs) (Carninci et al., 2005; Djebali et al., 2012; Dunham et al., 2012). NcRNAs are subdivided on basis of their length into small ncRNAs (<200 nucleotides; e.g. microRNAs (miRNAs), small interfering RNAs (siRNAs) and piwi‐interacting RNAs (piRNAs) and long ncRNAs (lncRNAs; >200 nucleotides), that differ in their biogenesis and function. LncRNAs show enormous functional diversity, influencing (post‐)transcriptional and translational diversification of specific genes and gene networks (Qureshi & Mehler, 2012; Wang & Chang, 2011). It is estimated that approximately 2% of the human genome is transcribed into lncRNAs (Managadze et al., 2013; Palazzo & Lee, 2015). LncRNAs are mainly transcribed by RNA polymerase II (Guttman et al., 2009) and based on their location in the genome can be classified into the following five categories: (a) sense lncRNAs, transcripts transcribed from the sense strand of protein‐coding genes and that contain one or more exons; (b) antisense lncRNAs, transcripts transcribed from the opposite strand of protein‐coding genes and that contain one or more exons; (c) bidirectional lncRNAs, transcripts transcribed from the opposite strand that share the same promoter as another gene and that are usually located <1,000 base pairs (bp) away from each other in close genomic proximity; (d) intronic lncRNAs, transcripts transcribed from intronic regions without showing overlap with exons; and (e) intergenic lncRNAs, independent transcripts located in between two genes (Derrien et al., 2012; Ma et al., 2013). Another interesting class of lncRNAs are circular RNAs (circRNAs). CircRNAs are produced by back‐splicing events that result in a closed loop structure, where a covalent bond is formed by linking 5’ (splice donor) and 3’ (splice acceptor) RNA splice sites. CircRNAs can be transcribed from both exonic and intronic regions, but the majority of circRNAs are exonic (Chen, 2016; Jeck et al., 2013; Memczak et al., 2013).

**Figure 1:**
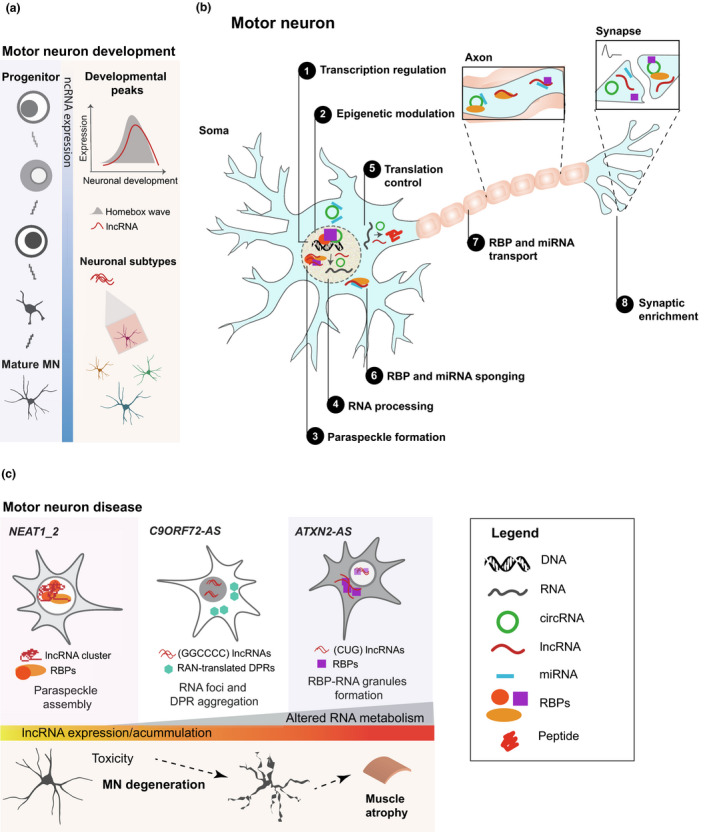
Schematic illustration highlighting the roles of long non‐coding RNAs (lncRNAs) in motor neuron (MN) development and disease. (a) Non‐coding RNA (lncRNA and circRNA) expression is enhanced during MN differentiation suggesting roles in the regulation of MN development from progenitor stages to mature motor neurons (MNs). An interesting link has been established between lncRNA expression and homeobox (*Hox*) gene expression. LncRNAs influence *Hox* gene expression but can also be derived from the *Hox* gene cluster. A role for lncRNAs in the specification of neuron subtypes has been proposed. (b) LncRNAs and circular RNAs have been implicated in a wide range of cellular and molecular functions in developing MNs, all of which (in)directly impact on gene expression regulation. LncRNAs have been reported to play a role in transcription regulation, epigenetic modulation, paraspeckle formation, RNA processing, translational control, miRNA sponging and synaptic enrichment of RNAs and proteins. CircRNAs have been reported to play a role in transcription regulation, RNA processing, RBP and miRNA sponging, RBP and miRNA transport and synaptic enrichment of RNAs and proteins. (c) Several lncRNAs have been linked to MN disease, three examples of which are shown here. Nuclear‐enriched abundant transcript 1 (*NEAT1)_2* is a lncRNA that regulates paraspeckle assembly and that clusters with several RNA‐binding proteins (RBPs) and inhibits their function. Changes in *NEAT1_2* and paraspeckle assembly in general may contribute to the pathogenesis of amyotrophic lateral sclerosis (ALS). *C9ORF72‐AS* is an antisense lncRNA that forms RNA foci and toxic dipeptide repeat (DPR) proteins in different cellular compartments because of the presence of hundreds to thousands hexanucleotide repeats in the 5’ region of the *C9ORF72* locus in C9‐ALS patients. In RNA foci *C9ORF72‐AS* aberrantly interacts with RBPs which may contribute to cellular toxicity. *ATAXIN2‐AS* is an antisense lncRNA that forms CUG repeat‐containing RNA aggregates recruiting RBPs leading to neurotoxicity. In ALS, patients may carry an expanded CAG repeat region in the 5’ coding part of the gene. Overall, lncRNA dysregulation may lead to altered RNA metabolism as a result of interference with RBPs and in case of C9‐ALS the accumulation of toxic DPR proteins. These defects contribute to MN degeneration and muscle atrophy as observed in ALS patients

Mechanistically, lncRNAs can act at the transcriptional and post‐transcriptional level to affect transcription, RNA processing and translation (Elling et al., 2016). Mediation of chromatin interactions, involvement in the formation of nuclear structures (e.g. paraspeckles), formation of decoys for transcription factors or as scaffolds for DNA‐binding proteins and RNA‐binding proteins (RBPs), functioning as miRNA sponges and control of mRNA decay are core functions of lncRNAs (Figure 1; Marchese et al., 2017; Quinn & Chang, 2016). Even though the majority of lncRNAs is present in the nucleus, some lncRNAs are found in the cytoplasm (Kung et al., 2013; Noh et al., 2018; Rinn & Chang, 2012; Ulitsky & Bartel, 2013). In the cytoplasm, lncRNAs are involved in processes such as RNA editing, RNA splicing and protein machinery assembly for translation initiation (Noh et al., 2018; Rashid et al., 2016). The majority of lncRNAs are of low abundance. In general, expression of lncRNAs is approximately 10‐fold lower as compared to protein‐coding transcripts (Cabili et al., 2011; Ravasi et al., 2006). This difference can in part be explained by prominent differences in lncRNA expression between cell types and tissues, and the strong spatiotemporal regulation of lncRNA expression as compared to mRNA expression (Cabili et al., 2011; Field et al., 2019; Yan et al., 2013; Yunusov et al., 2016). Ubiquitously expressed lncRNAs are generally expressed at high levels, while cell type‐ or tissue‐specific lncRNAs, such as those in MNs, are often expressed at lower levels (Jiang, Li,, et al., 2016). In addition to relatively low levels of expression, many lncRNAs show low levels of evolutionary and sequence conservation (Basu et al., 2013; Ulitsky et al., 2011). It has been suggested that evolutionary conserved lncRNAs may have important general functions, while species‐specific lncRNAs could play relevant roles in biological processes that are crucial for a specific species (e.g. expansion of the brain in primates). Most of the lncRNAs discussed in this review are conserved among mammals (Table 1).

**Table 1:** Evolutionary conservation of motor neuron lncRNAs

Name	ncRNA	Reported species	References
*Meg3*, *Rian* and *Mirg*	lncRNA	All mammals	Ogata and Kagami, (2016) and Yen et al., (2018)
*CAT7*	lncRNA	All mammals	Ray et al., (2016)
*Hoxb5os*	lncRNA	Mouse and human	Papaioannou et al., (2019)
*Gm12688/Gm14204*	lncRNA	Mouse, human (NA)	Rizvi et al., (2017)
*LncMN−1*,*−2*,*−3* and *Lhx1os*	lncRNA	Mouse and human	Biscarini et al., (2018)
*Lncrps25*	lncRNA	*Danio rerio* and human	Gao et al., (2020) and Ulitsky et al., (2011)
*Malat1*	lncRNA	All mammals	Ulitsky et al., (2011)
*Rmst*	lncRNA	Birds and mammals	Chodroff et al., (2010)
*Xist*	lncRNA	All mammals	Johnsson et al., (2014)
*Miat (Gomafu)*	lncRNA	All mammals	Chodroff et al., (2010); Sone et al., (2007)
*FUS‐linked circRNAs*	circRNA	Mouse and human	Errichelli et al., (2017)
*SMN circRNAs*	circRNA	Mouse and human	Ottesen et al., (2019)
*NEAT1*	lncRNA	Mouse and human	Clemson et al., (2009)
*BDNFOS*	lncRNA	Primates	Lipovich et al., (2012)
*TFEBα*	lncRNA	Human	Davis et al., (2003)
*Myolinc*	lncRNA	Mouse	Militello et al., (2018)
*SATIII*	lncRNA	*Drosophila melanogaster* and human	Chung et al., (2018)

Note

Overview of the reported conservation of lncRNAs discussed in this review. LncRNAs are listed according to their discussion in the main text.

Abbreviations: FUS, fused in sarcoma; Meg3, maternally expressed gene 3; NA, not annotated; SMN, survival motor neuron.

A remarkably large number of annotated lncRNAs, that is, approximately 40% (4,000 – 20,000 lncRNA genes), is expressed specifically in the brain (Derrien et al., 2012). In situ hybridization data from the Allen brain atlas show the expression of hundreds of lncRNAs in specific regions of the mouse brain, in specific types of neural cells and even in specific subcellular compartments. Several of these expression patterns are similar to those described for coding mRNAs, hinting at important neuronal functions for lncRNAs (Mercer et al., 2008). Indeed, lncRNAs have been linked to processes such as neuron development, neurite growth, synaptic transmission, memory consolidation and ageing (Derrien et al., 2012; Mehler & Mattick, 2007; Mercer et al., 2008; Pereira Fernandes et al., 2018; Shi et al., 2017). In this review, we specifically focus on the proposed role of lncRNAs in motor neurons (MNs). MNs are a group of neurons that have their cell bodies in the cortex (upper MNs) or in the brainstem and spinal cord (lower MNs) and project axons into the brainstem, spinal cord or towards peripheral muscles. These projections control essential functions such as movement, breathing and swallowing. Not surprising given their important functions, selective degeneration of MNs is a hallmark of motor neuron diseases (MNDs) such as amyotrophic lateral sclerosis (ALS) and spinal muscular atrophy (SMA). Here we review the role of lncRNAs in the development and functions of MNs, discuss how lncRNAs may contribute to MNDs and provide directions for future research.

## 2 LNCRNAS IN MOTOR NEURON DEVELOPMENT

### 2.1 Linear LncRNAs in developing motor neurons

MN development is complex and depends on the interplay between different molecular factors. Several lines of experimental evidence link lncRNAs to the development of MNs (Figure 1a,b, see also Table 2).

**Table 2:** Overview of the expression and proposed function of lncRNAs in motor neuron development

Name	ncRNA	Regulation	Observation	Mechanism	References
*Meg3*	lncRNA	Up‐regulated; spatial regulation	Regulated throughout embryonic stem cells–motor neuron (ESC‐MN) differentiation; enriched in the nucleus	Epigenetic regulation of *Hoxa4:Hoxc5* expression	Yen et al., (2018)
*CAT7*	lncRNA	Up‐regulated	Regulated during early stages of human ESC‐MN differentiation	Regulation of polycomb repressive complex 1 (PRC1) associated genes	Ray et al., (2016)
*Hoxb5os*	lncRNA	Up‐regulated	Regulated throughout ESC‐MN differentiation	Tbd	Rizvi et al., (2017)
*Gm12688/Gm14204*	lncRNA	Cell type‐specific expression	Uniquely expressed in V1/V1 and V2b GABAergic interneurons	Tbd	Rizvi et al., (2017)
*LncMN−1*,*−2*,*−3* and *Lhx1os*	lncRNA	Cell type‐specific expression; up‐regulated	Specifically enriched in MNs; regulated during differentiation of mouse ESC (mESC)/ human‐induced pluripotent stem cells (hiPSC)‐derived MNs	Tbd	Biscarini et al., (2018)
*Lncrps25*	lncRNA	Down‐regulated	Knockdown reduces swimming activity because of defects in primary MNs	Via *olig2* (Tbd)	Gao et al., (2020)
*Malat1*, *Meg3*, *Rmst*, *Xist* and *Miat*	lncRNA	Spatial distribution	Specifically enriched in somatodendritic/axonal fractions	Tbd	Briese et al., (2016)
*c−1*, *c−2*, *c−13*, *c−16*, *c−48*, *c−80*, *c−82*, *c−84*, *c−88*	circRNA	Up‐regulated	Regulated during mESC/hiPSC‐derived MN differentiation	Tbd	Errichelli et al., (2017)
*Human circSMN*	circRNA	Multiple isoforms produced	Primate specificity of SMN‐derived circRNAs	Tbd	Ottesen et al., (2019)

Abbreviations: hiPSC, human‐induced pluripotent stem cells; Meg3, maternally expressed gene 3; mESC, mouse embroynic stem cells; Tbd, to be determined.

### 2.2 *Meg3*

The imprinted mammalian *Dlk1‐Dio3* locus produces multiple lncRNAs from the maternally inherited allele. Profiling of differentiating embryonic stem cell (ESC)‐derived MNs revealed a predominant and gradual enrichment of maternally expressed gene 3 (*Meg3)* and other lncRNAs from the imprinted *Dlk1‐Dio3* locus (*Rian* and *Mirg*) at post‐mitotic stages of rostral MN development (Y. P. Yen et al., 2018). This enrichment was found not only in vitro in ESC‐MNs but also in vivo in mouse spinal cord. Interestingly, both the over‐expression of MN transcription factors and the treatment with retinoic acid in a normally maternally inherited methylated region context (*IG‐DMR^matΔ^* ESC) induced *Meg3* expression. Therefore, these factors, that are crucial for MN development, may drive *Meg3* expression during differentiation of ESCs into rostral brachial MNs. *Meg3* was found to be enriched in the nucleus of MNs while being chromatin‐associated. This strongly suggested a role in gene regulation. Indeed, by combining gene knockdown and chromatin immunoprecipitation, *Meg3* was shown to facilitate polycomb repressive complex 2 (PRC2)–Jarid2 interactions in post‐mitotic MNs. *Meg3* knockdown led to a global down‐regulation of H3K27me3 and rostral Homeobox (*Hox)* genes, and an up‐regulation of MN progenitor genes (*Pax6* and *Irx3*) and of caudal *Hox* genes (a *Hox5* to *Hoxc8* shift). *Hox* genes are transcription factors that regulate MN fate along the rostro‐caudal axis of the central nervous system (CNS). Based on these and other findings it was proposed that *Meg3* facilitates PRC2:Jarid2 complex formation to perpetuate rostral MN cell fate by maintaining the silenced epigenetic state of MN progenitor and caudal *Hox* genes (Y. P. Yen et al., 2018).

### 2.3 *CAT7*

Another screen designed to detect lncRNAs co‐precipitating with polycomb repressive complex 1 (PRC1) from chromatin identified multiple lncRNAs that influence the expression of polycomb group protein‐regulated genes. Loss of a novel lncRNA, *CAT7*, led to an increase in MN and pancreas homeobox 1 (MNX1 or HB9) expression in human embryonic stem cells‐derived MNs (Ray et al., 2016). MNX1 is a transcription factor that regulates the formation of pancreatic beta islets and MNs (Bonnefond et al., 2013; Van Arensbergen et al., 2010). During MN development, *MNX1* expression is silenced at early stages of differentiation as a result of PRC1 binding to the *MNX1* locus (Van Arensbergen et al., 2010). *CAT7* enhances the binding of PRC1 to the *MNX1* locus before activation of pathways that determine MN fate. *CAT7* shares high sequence similarity to a non‐syntenic *Danio rerio* (zebrafish) analog, *zcat7l*. Depletion of *zcat7l* by morpholino interference resulted in microcephaly at 48 hr post‐fertilization (hpf) and mortality starting on day 5. These phenotypes were successfully reverted by co‐expression of the human *CAT7* lncRNA isoform, indicating possible conserved functions between these non‐syntenic lncRNAs. The phenotypes found following *zcat7l* depletion were also observed following knockdown of the zebrafish polycomb complex proteins bmi1a/b, and *zcat7l* and *bmi1a/b* genetically interacted in combined knockdown experiments. Overall, these data indicate a functional link between *CAT7*/*zcat7l* lncRNAs and PRC1 in human and zebrafish. More generally, these findings show that lncRNAs can regulate and promote transcription activation at specific loci (Ray et al., 2016).

### 2.4 *Gm12688* and *Gm14204*

In another study, single‐cell topological RNA sequencing analysis was used to characterize the mechanisms underlying in vitro differentiation of mouse embryonic stem cell (mESC)‐derived MN cultures (Rizvi et al., 2017). Specific temporal regulation of lncRNAs was found during pluripotency, at the neural progenitor stage and during the transition to neuronal maturation. Different stages of MN differentiation are characterized by waves of *Hox* gene activation (Figure 1a). Intriguingly, these waves were accompanied by an up‐regulation of lncRNAs that originate from the opposite strand of these genes. This co‐expression is in line with previous reports identifying lncRNAs as regulators of *Hox* gene clusters (Dinger et al., 2008; Ponjavic et al., 2009; Rinn et al., 2007; Wang et al., 2011). In addition, several lncRNAs were detected in other neuron types in the cultures, which contain MNs and concomitant cell types (i.e. those found in the spinal cord). For example, *Gm12688*, an intergenic lncRNA located near *Foxd3* and transcribed from the opposite strand, was found in V1 interneurons. *Gm14204*, an intergenic lncRNA located near *Solute carrier family 32 member 1* (*Slc32a1)* and transcribed from the opposite strand, was restricted to V1 and V2b GABAergic interneurons (Rizvi et al., 2017). The discovery of these intergenic lncRNAs suggests that lncRNAs may play a role in neuronal diversification in the spinal cord.

### 2.5 *Lhx1os*, *Lnc‐MN1*, *LncMN‐2* and *LncMN‐3*

Stage‐specific expression of lncRNAs was also detected in an analysis of the long non‐coding transcriptome of mESC‐ and human‐induced pluripotent stem cells (hiPSC)‐derived MNs purified by fluorescence‐activated cell sorting (FACS). The expression of several lncRNAs was restricted to MNs, as compared to absence of signal in green fluorescent protein‐negative cells (i.e. MN progenitors and interneurons). Interestingly, these lncRNAs showed a predominant cytoplasmic expression (Biscarini et al., 2018). Expression of a subset of mouse lncRNAs (*Lhx1os*, *2610316D01Rik*, *5330434G04Rik* and *A730046J19Rik*) was specifically increased at later stages of ESC‐MN development (from day 4 onwards), during which MN progenitors develop into post‐mitotic MNs and differentiate further. This lncRNA cluster was conserved in human (renamed *Lhx1os*, *Lnc‐MN1*, *LncMN‐2* and *LncMN‐3*) and showed a similar mode of expression regulation during hiPSC‐derived MN development. These results further confirm the role of lncRNAs in MN differentiation and hint at strong evolutionary conservation of the mechanism‐of‐action and function of lncRNAs in MNs.

### 2.6 *Malat1*, *Meg3*, *Rmst*, *Xist* and *Miat*

In addition to studies reporting general changes in lncRNA expression, other work has focused on the spatial enrichment of these non‐coding RNAs in specific subcellular compartments. Initially, lncRNAs had predominantly been detected in the nucleus and were therefore studied for potential roles in the regulation of gene expression (Sun et al., 2018; Ulitsky & Bartel, 2013). The presence of lncRNAs in other subcellular compartments hinted at the exciting possibility of additional and different molecular functions. Briese et al. (2016) investigated the abundance of several lncRNAs in the axonal or somatodendritic compartment, including that of metastasis‐associated lung adenocarcinoma transcript 1 (*Malat1)*, *Meg3*, rhabdomyosarcoma 2‐associated transcript (*Rmst)*, X‐inactive‐specific transcript (*Xist)* and Myocardial infarction‐associated transcript (*Miat)*. While some lncRNAs showed similar levels in different compartments, *Meg3* and *Rmst* were enriched in somatodendritic and axonal fractions, respectively (Briese et al., 2016). The molecular mechanisms dictating this highly specific distribution are unknown. However, a number of lncRNAs can interact with proteins and it is plausible that protein:RNA complexes play a role in compartment‐specific lncRNA distribution (Briese et al., 2016; Quan et al., 2017).

### 2.7 *Lncrps25*

While many of these studies reveal interesting lncRNA expression patterns that hint at roles during MN differentiation, recent work has also begun to explore the functional role of lncRNAs in MNs in vivo. For example, the lncRNA *lncrps25* was found to be strongly expressed in the embryonic zebrafish CNS and co‐expressed with *mnx1*, while *lncrps25* knockdown induced body abnormalities and striking changes in locomotor behaviour, that is, decreased swimming distance and speed (Gao et al., 2020). These phenotypes were caused by defects in primary MNs that showed reduced axon length and branching. *Lncrps25* depletion led to a reduced expression of oligodendrocyte transcription factor 2 (*olig2*) in brain and spinal cord. *Olig2* co‐expression could partially rescue the motor phenotypes caused by *lncrps25* depletion. Olig2 is part of the *Olig* gene family that encode for basic helix‐loop‐helix transcription factors that are required for MN and oligodendrocyte development (Lu et al., 2002; Park et al., 2002). Based on the effect of *lncrps25* depletion, and *lncrps25* and *olig2* expression patterns, it was proposed that *lncrps25:*olig2 interactions occur at the neural plate stage to eventually affect MN development (Gao et al., 2020). However, the precise mechanistic details of this lncRNA–mRNA pathway remain to be defined. A recent study focusing on defining lncRNA networks in oligodendrocytes identified the oligodendrocyte‐specific lncRNA *LncOL1*. *LncOL1* is involved in oligodendrocyte differentiation and CNS (re)myelination (He et al., 2017). Interestingly, a peak in genomic occupancy by Olig2 was detected around the transcription start site of *LncOL1*, suggesting a role for Olig2 in the transcriptional regulation of *LncOL1*. Therefore, Olig2 appears to be a shared factor in the regulation of MN and oligodendrocyte differentiation through interactions with different lncRNAs (*lncrps25* and *LncOL1*). This is intriguing since the specification of MN and oligodendrocytes from ventral progenitor cells is a sequential process mediated by olig2 (Ravanelli & Appel, 2015). Further, oligodendrocytes are known to play a role in MNDs such as ALS. Oligodendrocyte dysfunction contributes to MN excitability and death, and consequently to subsequent oligodendrocyte‐related pathology (Ferraiuolo et al., 2016). It will be interesting to determine how Olig2:lncRNA networks interact during MN and oligodendrocyte development, and how their dysfunction may lead to disease.

### 2.8 CircRNAs in developing motor neurons

CircRNAs are highly enriched in the brain, dynamically regulated and conserved across species (Rybak‐Wolf et al., 2015). The mechanisms that cause brain‐enriched circRNA expression remain to be determined. One explanation is that many circRNAs derive from genes that are enriched in brain tissue, but the existence of neuron‐specific biogenesis mechanisms has also been proposed (van Rossum et al., 2016). Another interesting observation is that whereas linear lncRNAs often display cell type‐specific expression (e.g. in MNs), cell type‐specific circRNAs are rare. This difference may arise from the fact that whereas many lncRNAs originate from intergenic stretches of the genome that lack protein‐coding genes, circRNAs are generally produced from protein‐coding transcripts (Memczak et al., 2013; Ransohoff et al., 2018; Salzman et al., 2012). MN‐specific circRNAs therefore mainly arise from MN‐specific genes, while the intergenic regions that produce other lncRNAs may be susceptible to different kinds of (post)transcriptional regulation. CircRNAs are differentially expressed across brain regions, subcellular compartments and regulated at pre‐ and postnatal stages (Chen & Schuman, 2016; Memczak et al., 2013; Rybak‐Wolf et al., 2015; Venø et al., 2015; You et al., 2015). Many of these RNA molecules derive from genes that encode for synaptic proteins or proteins with essential roles in neural development. Further, the expression of several circRNAs is influenced by neuronal activity and they are shown to accumulate during ageing (Venø et al., 2015; Westholm et al., 2014). Together, these observations hint at roles for circRNAs in a wide range of neuronal processes in brain development, function and disease (van Rossum et al., 2016).

The functional roles and mechanism‐of‐action of most circRNAs remain poorly understood. A detailed discussion of the function of circRNAs is beyond the scope of this review, but several other recent reviews cover our current knowledge of the molecular effects and functional roles of circRNAs (Ebbesen et al., 2016; Meng et al., 2019; van Rossum et al., 2016; Salzman, 2016; Sekar & Liang, 2019). Translation into small peptides, miRNA sponging and sequestration of RBPs are some of the currently accepted mechanisms by which circRNAs can act (Li et al., 2018).

### 2.9 FUS‐linked circRNAs

CircRNAs have also been detected in MNs. Hundreds of circRNAs were found in mESC‐derived MNs and, similar to that has been demonstrated for other lncRNAs, their expression increased during MN differentiation (Errichelli et al., 2017). The majority of identified circRNAs was conserved in hiPSC‐derived MNs. In addition to expression profiling, this study also provided insight into the mechanisms underlying circRNA biogenesis in MNs. The ALS‐associated protein fused in sarcoma (FUS) was shown to promote circRNA formation in MNs without affecting their linear counterparts. FUS is an RBP with roles in RNA splicing and may contribute to circRNA formation by regulating the back‐splicing events that lead to RNA circularization. In line with this idea, cross‐linking immunoprecipitation (CLIP) assays revealed a specific enrichment of FUS at circularizing exon–intron regions of selected circRNAs (*c‐1*, *c‐2*, *c‐13*, *c‐16*, *c‐48*, *c‐80*, *c‐82*, *c‐ 84*, *c‐88*) (Errichelli et al., 2017; Verheijen & Pasterkamp, 2017).

### 2.10 SMN circRNAs

Although the biogenesis and functions of circRNAs are often evolutionarily conserved, in some instances the abundance, the presence of specific isoforms or functions of circRNAs are species‐specific (Rybak‐Wolf et al., 2015). Here, it is worth mentioning the human *survival motor neuron* (*SMN*) locus, which is able to originate a surprisingly large number of different exonic circRNAs (Ottesen et al., 2019). SMN protein is essential for MN development and survival and its loss causes SMA (Chaytow et al., 2018; Fallini et al., 2012; Lefebvre et al., 1995). Most of the reported functions of SMN are linked to RNA processing. However, it is known that SMN protein accumulates in the axons of MNs during development hinting at roles in, for example, local gene expression regulation (Gabanella et al., 2005; Giavazzi et al., 2006; Groen et al., 2013; Zhang et al., 2006). In addition to SMN protein, the *SMN* locus also generates a vast array of circRNAs. Using intron–exon site complementarity analysis in MN‐like NSC‐34 cells (mouse MN‐like hybrid cell line), the mouse *Smn* locus was predicted to give rise to a considerably lower number of circRNAs as compared to the human *SMN* locus (Ottesen et al., 2019). These differences seem to result from a lower number of short interspersed nuclear elements in the intronic regions of the mouse *Smn* gene. A substantial number of *SMN*‐derived circRNAs was specific to primates. It is therefore possible that specific circRNAs may be directly linked to MN functions that have become more important during evolution. The consequence of the species‐specific expression of *SMN*‐derived circRNAs and the functional roles of these lncRNAs remain to be determined. Nevertheless, this insight highlights the need for more knowledge on how genetic differences influence non‐coding RNA biogenesis and function.

## 3 LNCRNAS IN MOTOR NEURON DISEASE

LncRNAs are strongly regulated by exposure to drugs, environmental factors (e.g. toxins) or as a consequence of pathological situations. In many instances, regulation of lncRNA expression confers neuroprotection or induces anti‐apoptotic responses (Table 3). Given this regulation of lncRNAs, their dynamic expression patterns in MNs and their emerging roles in MN development and function, it is not surprising that lncRNA dysregulation has been implicated in MNDs. Understanding the mechanisms‐of‐action and functions of lncRNAs may assist the development of new therapies for MNDs. Here, we discuss and summarize the expression and functional role of lncRNAs in different MN‐related diseases (see also Table 4), extending on previous reviews (Chen & Chen, 2020; Gagliardi, et al., 2018).

**Table 3:** Expression changes in motor neuron lncRNAs in response to exposure to external factors or drugs

LncRNA	Treatment/stimulus	Response	(Neuro) protection or Disease onset	System	References
*Meg3*	5‐aza−2‐deoxycytidine (demethylating agent)	Up‐regulation	Tbd	Human breast cancer tumour cell lines; Human meningioma cell line; human hepatocellular cancer (HCC) cell lines	Braconi et al., (2011); Zhang et al., (2010) and Zhao et al., (2005)
	Hypoxia	Up‐regulation	Tbd	Human umbilical vein endothelial cells (HUVECs)	Michalik et al., (2014)
	Serum starvation	Up‐regulation	Tbd	Mouse embryonic fibroblasts (MEFs)	Wang, Liang, et al. (2019)
	Heroin	Up‐regulation	Tbd	Human nucleus accumbens (NAc)	Michelhaugh et al., (2011)
*Hoxb5os*	Serum starvation	Down‐regulation	Tbd	MEFs	Wang, Liang, et al. (2019)
*Malat1*	Polycyclic aromatic hydrocarbons (PAHs) exposure	Up‐regulation	Tbd	Peripheral blood lymphocytes (PBLCs) of male coke oven workers	Gao et al., (2016)
	Hypoxia	Up‐regulation	Tbd	HUVECs; Several tissues of mice exposed to hypoxia (including brain)	Lelli et al., (2015); Michalik et al., (2014)
	X‐irradiation	Up‐regulation	Tbd	Human lymphoblast cell line TK6	Chaudhry, (2014)
	β‐Asarone	Down‐regulation	Neuroprotective	In vitro and in vivo (mice)	Zhang et al., (2016)
	doxorubicin (DOX), 5‐fluorouracil (5‐FU), Mitomycin	Up‐regulated	Anti‐apoptotic	DOX, 5‐FU and Mitomycin‐resistant HCC cells	Yuan et al., (2016) and Zhang et al., (2020)
	Heroin	Up‐regulation	Tbd	Human NAc	Michelhaugh et al., (2011)
*Rmst*	X‐irradiation	Up‐regulation	Tbd	Human lymphoblast cell line TK6	Chaudhry, (2014)
*Xist*	DOX	Up‐regulation	Tbd	DOX‐resistant colorectal cancer tissues and cells	Zhu et al., (2018)
*Miat (Gomafu)*	Cisplatin (CDDP)	Up‐regulation	Tbd	(CDDP)‑resistant H1299 cells	Wu et al., (2020)
	Potassium chloride (KCl)	Down‐regulation	Might contribute to alternative splicing observed in schizophrenia (SCZ)	Neuronal stimulation of mouse primary cortical neurons and hiPSC‐derived neurons. Reduced expression in SCZ patient tissue	Barry et al., (2014)
	Serum starvation	Down‐regulation	Tbd	MEFs	Wang, Liang, et al. (2019)
	Heroin and Cocaine	Up‐regulation	Tbd	Human NAc	Albertson et al., (2006) and Michelhaugh et al., (2011)
*Neat1*	Hypoxia	Up‐regulation	Tbd	Several tissues of mice exposed to hypoxia (including brain). In vitro and in vivo (mice)	Lelli et al., (2015)
	Serum starvation	Up‐regulation		MEFs	Wang, Liang, et al. (2019)
	LPS	Up‐regulation	KD improves protection against LPS‐induced myocardial injury.	In vivo (mice)	Wang, Liu, et al. (2019)
	H_2_O_2_ induced (oxidative stress)	Up‐regulation	Increased viability. May contribute to neuroprotection in Huntington's disease	N2A cells	Sunwoo et al., (2017)
	Heroin	Up‐regulation	Tbd	Human NAc	Michelhaugh et al., (2011)
	KCl	Up‐regulation	Tbd	SH‐SY5Y cells	Lipovich et al., (2012)
	Fenofibrate and Simvastatin	Up‐regulation	Neuroprotective	SH‐SY5Y cells	Simchovitz et al., (2019)
*BDNFOS*	KCl depolarization	Down‐regulation	Negative regulator of brain‐derived neurotrophic factor (BDNF)	SH‐SY5Y cells	Lipovich et al., (2012)

Note

Overview of the expression changes in lncRNAs in response to various stimuli and their proposed effects that are discussed in this review.

Abbreviations: HCC, hepatocellular cancer; hiPSC, human‐induced pluripotent stem cells; LPS, lipopolysacharide; *Malat1*, metastasis‐associated lung adenocarcinoma transcript 1; MEFs, mouse embryonic fibroblasts; Meg3, maternally expressed gene 3; Tbd, to be determined.

**Table 4:** Overview of the expression and proposed functions of lncRNAs in motor neuron disease

Name	ncRNA	Disease	Regulation	Function	References
*NEAT1*	lncRNA	ALS	Up‐regulated at early stage	Regulates paraspeckle formation, increased *NEAT1* expression leads to neurotoxicity	Clemson et al., (2009); Nishimoto et al., (2013) and Suzuki et al., (2019)
*C9ORF72‐AS*	antisense RNA	ALS	Up‐regulated	Forms RNA foci that recruit RBPs, DPR protein formation via repeat‐associated non‐ATG‐initiated (RAN) translation leading to neurotoxicity	Cheng et al., (2019); Mizielinska et al., (2014); Mori, et al. (2013); Sareen et al., (2013); Swinnen et al., (2018) and Wen et al., (2014)
*ATXN2‐AS*	antisense RNA	ALS	Up‐regulated	Repeat expansion RNA induces neurotoxicity	Li, Sun, et al. (2016)
*SMN‐AS*	antisense RNA	SMA	Up‐regulated	Recruits polycomb repressive complex 2 (PRC2) complex to the *SMN* gene to suppress SMN expression	d’Ydewalle et al., (2017) and Woo et al., (2017)
*ZEB1‐AS*, *ZBTB11‐AS*	antisense RNA	ALS	Up‐regulated in blood samples (peripheral blood mononuclear cells [PBMCs])	Tbd	Gagliardi, et al. (2018)
*UBXN7‐AS*, *ATG10‐AS*, *ADORA2A‐AS*	antisense RNA	ALS	Up‐regulated in blood samples (PBMCs)	Tbd	Gagliardi, et al. (2018)
*hsa_circ_0001173*, *hsa_circ_0043138*, *hsa_circ_0088036*	circRNA	ALS	Up‐regulated in blood samples (PBMCs)	Biomarker potential	Dolinar et al., (2019)

Abbreviations: ATXN2, Ataxin‐2; NEAT1, nuclear‐enriched abundant transcript 1; SMN, survival motor neuron; Tbd, to be determined.

### 3.1 Amyotropic lateral sclerosis (ALS)

ALS is a fatal disease characterized by the progressive loss of MNs in brain and spinal cord. About 90%–95% of cases lack a clearly identifiable hereditary or environmental cause classically being referred to as sporadic ALS (sALS). About 5%–10% of cases are defined as familial ALS (fALS) because of demonstration of the direct inheritance (Renton et al., 2014), although the simple distinction between sALS and fALS is no longer tenable (Al‐Chalabi et al., 2016). In most countries, the prevalence of ALS is approximately four to six cases per 100,000 people, with a median age of onset of 65 years for sALS, whereas this is 10 years earlier for fALS cases (Al‐Chalabi & Hardiman, 2013; van Es et al., 2017). As disease progresses, a large proportion of upper (corticospinal) MNs, projecting from the motor cortex to the brainstem and spinal cord, and bulbar and spinal MNs, projecting to skeletal muscles, degenerate. This ultimately leads to spasticity and muscle atrophy which eventually results in weakness and paralysis. Eventually, patients die as a result of paralysis of respiratory muscles within 3–5 years after symptom onset (van Es et al., 2017).

The causative mechanisms that lead to MN degeneration and ALS remain incompletely understood. Mutations in more than 30 genes have been linked to ALS, and based on the functions of these genes, multiple disease pathways have been proposed and investigated. The most commonly mutated genes are *chromosome 9 ORF 72* (*C9ORF72*; hexanucleotide (*GGGGCC*)*_n_* (*G_4_C_2_*) repeat expansion in a non‐coding genomic region), superoxide dismutase1, TAR DNA‐binding protein and *FUS* (Renton et al., 2014). These genes regulate molecular pathways controlling key cellular events such as RNA biology, protein turnover and axonal transport (Burk & Pasterkamp, 2019; Robberecht & Philips, 2013).

The RBPs TAR DNA‐binding protein 43 (TDP‐43) and FUS are normally located in the nucleus and influence RNA metabolism. In ALS MNs, an abnormal accumulation of these proteins is observed in the cytoplasm that is thought to contribute to MN degeneration because of effects on RNA processing and other RNA‐related mechanisms (Blokhuis et al., 2013). Further, the ALS‐associated hexanucleotide repeat expansion in the *C9ORF72* locus causes mutant *C9ORF72* pre‐mRNA to accumulate in RNA foci leading to accumulation of RBPs (Gendron et al., 2013; Renton et al., 2014). Given these and other data supporting a prominent role for defects in RNA biology in ALS, it is not surprising that lncRNAs also contribute to the development of ALS and other MNDs. Here we discuss a few lncRNAs that have been implicated in the pathogenesis of ALS or SMA.

### 3.2 *NEAT1*

*NEAT1* is a well‐characterized lncRNA that organizes nuclear structures called ‘paraspeckles’. Paraspeckles contain proteins involved in transcription and RNA processing (Clemson et al., 2009; Fox & Lamond, 2010). So far, two isoforms of *NEAT1*, *NEAT1_1* and *NEAT1_2*, have been described that are spliced by alternative processing of the 3‘‐untranslated region (Fox et al., 2018; Naganuma et al., 2012; Yamazaki et al., 2018). *NEAT1* is classified as one of the architectural RNAs (arcRNA), that function by providing essential scaffolds for the formation of nuclear bodies, with *NEAT1_2* playing a predominant role in paraspeckle formation (Chujo et al., 2016; Naganuma et al., 2012). Paraspeckle formation is a tightly regulated process that occurs parallel to *RNApolII* transcription of *NEAT1_2* and binding of several paraspeckle proteins (PSPs) to *NEAT1* (Mao et al., 2011; Yamazaki et al., 2018). So far, more than 60 PSPs have been identified, most of which are RBPs (Yamazaki & Hirose, 2015). RNAi studies identified seven essential PSPs that are able to form a paraspeckle with ~ 50 *NEAT1_2* molecules (Chujo et al., 2017; Naganuma et al., 2012). Six of the seven essential PSPs possess prion‐like‐domains, of which FUS and RNA‐binding motif protein 14 are shown to be required to form paraspeckles in vivo (Hennig et al., 2015; Yamazaki & Hirose, 2015). Functionally, PSPs interact with specific domains of *NEAT1* in the formation of paraspeckles. These domains were elucidated by systemic deletion of parts of *NEAT1_2* using CRISPR/Cas9‐mediated genome editing, revealing domains important for: 1) RNA stabilization of *NEAT1_2* (C‐terminal domain and N‐terminal TH structure), 2) paraspeckle assembly (middle domain) and 3) isoform switching (polyadenylation signal containing domain; Yamazaki et al., 2018). The middle domain of *NEAT1_2* further contains redundant functional subdomains responsible for paraspeckle assembly. By performing CLIP‐seq it was confirmed that PSPs non‐PoU domain‐containing octamer‐binding protein (NONO), splicing factor proline/glutamine‐rich (SFPQ) and FUS bind several of the *NEAT1_2* subdomains. Paraspeckle assembly could be rescued by artificial tethering of a *NEAT1_2* mutant lacking functional subdomains with one of the PSPs (NONO, SFPQ or FUS). The phase‐separation property of paraspeckles was shown by treatment with 1,6‐hexanediol, which not only disrupted paraspeckle structure but also disturbed interactions between NONO and RBPs that contain prion‐like‐domains like FUS. It is speculated that FUS through its PLD recruits several PSPs, leading to the formation of FUS oligomers thereby mediating paraspeckle assembly via phase separation (Yamazaki et al., 2018). Overall, these studies highlight the key role of *NEAT1_2* subdomains in paraspeckle assembly through phase separation.

Several lines of experimental evidence suggest that the size and number of paraspeckles is influenced by the expression level of *NEAT1*. Physiologically, *NEAT1* and paraspeckles are not detectable in human embryonic stem cells, but differentiation leads to the induction of *NEAT1* expression and formation of paraspeckles. Knockdown of *NEAT1* in HeLa cells leads to the loss of paraspeckles and increased nucleocytoplasmic export of mRNAs bearing *Alu* repeats, implicating *NEAT1* function in mRNA export (Chen & Carmichael, 2009). Induction of *NEAT1* levels concomitant with paraspeckle enlargement has been observed under different conditions, for example, during viral infection and myotube differentiation (Sunwoo et al., 2009; Zhang et al., 2013). Further, proteasome inhibition in HeLa cells by MG132 led to strong up‐regulation of *NEAT1* together with the elongation of paraspeckles and sequestration of the transcription factor SFPQ by *NEAT1*. In contrast, *NEAT1* knockout mouse embryonic fibroblasts display the loss of paraspeckles and are more susceptible to proteasome inhibitor‐mediated cell death, suggesting a pro‐survival role for paraspeckles under stressful conditions (Hirose et al., 2014).

As indicated above, *NEAT1* interacts with several RBPs involved in ALS (i.e. FUS and TDP‐43), both in human brain tissue and in cultured cells (Nishimoto et al., 2013; Tollervey et al., 2011). In early onset ALS patient tissue, not only the frequency of paraspeckle formation is highly increased but also the co‐localization of *NEAT1* with TDP‐43 and FUS in paraspeckles is enhanced. Further, by performing interaction and localization studies it was concluded that *NEAT1* acts as a scaffold for these RBPs in the nuclei of ALS MNs (Nishimoto et al., 2013). Subsequent work confirmed these observations, showing an increase in paraspeckle formation in the spinal cords of sALS and fALS patients (Shelkovnikova et al., 2018). ALS‐associated RBPs not only interact with *NEAT1* but also regulate *NEAT1* expression. Deletion of FUS, TDP‐43 or Matrin 3 (MATR3) leads to increased *NEAT1_2* RNA levels (Banerjee et al., 2017; Naganuma et al., 2012). Paraspeckles are known to exert anti‐apoptotic activity and can increase cell viability under stressful conditions (Hirose et al., 2014; Shelkovnikova et al., 2018). Their increase at early disease stages in ALS could hint at compensatory mechanisms to increase MN survival. Mutations in FUS lead to impaired paraspeckle formation by dysregulating *NEAT1* transcription and mis‐assembly of other paraspeckle proteins that are required during adequate stress responses (Shelkovnikova et al., 2014). This disrupts neuroprotective mechanisms and may contribute to the aggressive disease phenotype (early onset and fast progression) observed in FUS‐ALS cases (An et al., 2019). Although these studies suggest that ALS‐related RBP mutations affect *NEAT1* expression and may thereby influence paraspeckle formation, the precise role of *NEAT1* in ALS pathogenesis is incompletely understood. A recent study shows that the activation and up‐regulation of endogenous *NEAT1* levels using the CRISPR‐Cas9 system in NSC‐34 cells induces neurotoxicity. *NEAT1_2*, but not *NEAT1_1*, was proposed to mediate this neurotoxic effect (Suzuki et al., 2019). These data support the idea that altered *NEAT1* expression in ALS leads to defects in paraspeckle formation causing cell death and neurodegeneration (Figure 1c). However, a neuroprotective role for *NEAT1* is proposed in Parkinson's disease (PD) (Simchovitz et al., 2019). Increased *NEAT1* levels were found in the substantia nigra of PD patients and in drug‐induced oxidative stress in vitro PD models. This increase in expression was also associated with formation of *NEAT1* paraspeckles both in vitro and in vivo. Further, neuroprotective agents, like fenofibrate and simvastatin, were found to induce *NEAT1* expression, whereas *NEAT1* depletion increased oxidative stress‐induced cell death (Simchovitz et al., 2019).

Several of the genes associated with ALS give rise to sense or antisense RNAs which are not translated into protein but act as lncRNAs. These include naturally occurring (anti)sense RNAs but also RNAs that gain lncRNA functions as a result of genetic changes (e.g. repeat expansions). A few prominent examples are discussed below.

### 3.3 *C9ORF72*

As indicated in previous sections, the expansion of a repeat region of six‐nucleotide motifs (*GGGGCC*)*_n_* (*G_4_C_2_*) in the 5’ region of the *C9ORF72* gene is the most common genetic cause of ALS and frontotemporal dementia (FTD; DeJesus‐Hernandez et al., 2011; Renton et al., 2011; Zu et al., 2013). FTD is a form of dementia characterized by impaired judgement and executive skills, and loss of neurons in the frontal and temporal cortices (Burrell et al., 2016). ALS and FTD show overlapping disease phenotypes both at the symptomatic and genetic levels. The (*G_4_C_2_*) repeat expansions in *C9ORF72* are observed in three transcript variants: in variants 1 and 3 the expansions are located in intron 1 and in variant 2 they are present in the promoter region (Balendra & Isaacs, 2018). These *C9ORF72* repeat expansions are not only found in almost 40% of familial ALS and FTD cases, but also in up to 8% of sporadic cases of ALS and FTD (DeJesus‐Hernandez et al., 2011; Majounie et al., 2012; Rademakers, 2012; Renton et al., 2011). Healthy individuals carry up to 20–30 copies of the repeat, whereas repeat size increases to hundreds to thousands of (*G_4_C_2_*) sequences in ALS patients (DeJesus‐Hernandez et al., 2011). *C9ORF72* loss‐of‐function and toxic gain‐of‐function mediated by the repeat expansions have been implicated in C9ORF72‐associated ALS cases (C9‐ALS).

In addition to different sense isoforms of *C9ORF72*, one or more antisense transcripts that arise from the same promoter have been detected. The mutant hexanucleotide repeat regions of C9ORF72 are transcribed in a bidirectional fashion, producing sense (*C9ORF72‐S*), with *G_4_C_2_* repeats, and antisense (*C9ORF72‐AS*), with *GGCCCC* (*G_2_C_4_*) repeats, RNAs (Donnelly et al., 2013; Mizielinska et al., 2013; Mori, et al., 2013; Renton et al., 2011). Both expanded repeat‐containing transcripts (sense and antisense) can be translated into poly‐dipeptides (dipeptide repeat [DPR] proteins) which accumulate in MNs of C9‐ALS patients (Figure 1c; Freibaum & Taylor, 2017; Gendron et al., 2017; Mori, et al., 2013; Saberi et al., 2018; Wen et al., 2014; Zu et al., 2013). Several studies have focused on understanding the function of *C9ORF72‐S* RNA and its associated proteins (reviewed in Moens et al., (2017)). Over‐expression of *G_4_C_2_* repeats in cell culture, *C. elegans*, *Danio rerio* and *Drosophila melanogaster* led to deleterious phenotypes, which provided evidence that *C9ORF72‐*associated toxic gain‐of‐function contributed to the disease (Burguete et al., 2015; Freibaum et al., 2015; Kramer et al., 2016; Lee et al., 2013; Mizielinska et al., 2014; Wen et al., 2014; Xu et al., 2013; Zu et al., 2011). Similarly, adeno‐associated virus‐mediated over‐expression of 66 *G_4_C_2_* repeats in mice led to an accumulation of RNA foci and DPR proteins together with neuronal cell loss, TDP‐43 pathology and cognitive and motor dysfunction (Chew et al., 2015). The functional relevance of *C9ORF72‐AS* is less well understood. Interestingly, *C9ORF72‐AS* RNAs harbour a few miRNA‐binding sites in the first exon (Douglas, 2018). Similar to *C9ORF72‐S*, *C9ORF72‐AS* forms RNA foci in MNs and sporadically in interneurons (in frontal cortex and spinal cord) of ALS and FTD patients (DeJesus‐Hernandez et al., 2011; Gendron et al., 2013; Mizielinska et al., 2013; Zu et al., 2013). However, a higher frequency of *C9ORF72‐AS* as compared to *C9ORF72‐S* RNA foci and DPR proteins is observed in MNs of C9‐ALS patients (Cooper‐Knock et al., 2015). *C9ORF72‐AS* RNA foci are found in the peri‐nucleolar regions, suggesting that nucleolar defects or stress may contribute to C9‐ALS pathogenesis (Kwon et al., 2014; Tao et al., 2015). It is important to note, however, that RNA foci formed by either *C9ORF72‐S* or *C9ORF72‐AS* do not correlate with the extent of neurodegeneration observed in ALS patients and are not determining factors of the clinico‐pathological variability observed in C9‐ALS cases (DeJesus‐Hernandez et al., 2017). This may imply a role for other toxic modifiers in disease pathogenesis.

*C9ORF72‐S* expansions are reported to form G‐quadruplexes, which regulate transcription and gene expression (Fratta et al., 2012; Haeusler et al., 2014; Reddy et al., 2013; Wang, Goodrich, et al., 2019). *C9ORF72‐AS* repeats do not form G‐quadruplexes but can form i‐Motifs and hairpin structures, that can affect genome stability and transcription (Assi et al., 2018; Kovanda et al., 2015). Further, the crystal structure of *C9ORF72‐AS* repeat RNA showed that *C9ORF72‐AS* RNA forms a double helix structure with tandem C:C mismatches, that could attract proteins recognizing these structures (Dodd et al., 2016). Therefore, repeat expansions in *C9ORF72* may not only affect ALS pathogenesis through the formation of RNA foci or production of DPR proteins, but also indirectly by regulating the secondary structure of DNA and RNA, and thereby gene expression.

To determine how ALS‐associated *C9ORF72* repeat expansions cause ALS, different in vivo models have been generated. Expression of *C9ORF72* repeat expansions that form DPR proteins in *Drosophila* is toxic leading to adult‐onset neurodegeneration with reduced lifespan (Mizielinska et al., 2014). Expression of repeat expansion RNA that is not translated into DPR proteins did not induce neurotoxicity, suggesting that the toxic effect of *C9ORF72* repeat expansions may be mediated by DPR proteins (Mizielinska et al., 2014). To better understand the role of *C9ORF72‐S* and *C9ORF72‐AS* repeat RNA expansions, another study created RNA‐only *Drosophila* models, to avoid the effects of DPR proteins. Flies were generated to express 1) small (~100) RNA repeats in sense or antisense directions either as a processed polyadenylated transcript (named polyA) or as intronic versions fused with green fluorescent protein exons, or 2) large RNA‐only repeats (>1,000) in the sense direction (Moens et al., 2018). Both, sense and antisense polyA repeat RNA was found to accumulate in the cytoplasm of adult *Drosophila* neurons, whereas intronic RNAs with small and large repeats accumulated in the nucleus forming RNA foci, mimicking pathological conditions. Even though the RNA foci sequestered endogenous *Drosophila* RBPs, no neurodegenerative phenotypes or toxicity were observed following either sense or antisense RNA expression (small and large repeats). This suggests that mutant RNA is not sufficient to induce disease‐relevant toxicity (Moens et al., 2018). However, expression of repeat expansions in a *Danio rerio* (zebrafish) model induced neurodegenerative phenotypes (Swinnen et al., 2018). Injection of ~70 *C9ORF72‐S* (G_4_C_2_) or *C9ORF72‐AS* (G_2_C_4_) repeats in zebrafish embryos induced motor axonopathy. A dose‐dependent effect was observed on axon outgrowth and branching. Further, it was shown that toxicity induced by repeat RNA was independent of DPR proteins, suggesting that the presence of repeat RNA in the absence of DPR proteins is sufficient to induce MN toxicity in zebrafish (Swinnen et al., 2018). These differences in sensitivity for RNA‐induced toxicity may be explained by species differences or differences in experimental approaches. However, even within one species (mouse) different effects of the expression of repeat RNAs were found. Even though in most models RNA foci and DPR protein inclusions formed from both sense and antisense *C9ORF72* RNAs were observed, some models developed motor or cognitive defects, whereas others were unaffected (Jiang, Zhu, et al., 2016; Liu et al., 2016; O’Rourke et al., 2015; Peters et al., 2015). Further work is needed to carefully assess the role of RNA toxicity in C9‐ALS. Expression of longer repeat sequences (in the pathological range) may be required to model RNA toxicity accurately.

Despite this recent progress, the precise effects of the RNA foci or DPR proteins observed in C9‐ALS are still unclear. Although valuable, studies on post‐mortem tissue from patients do not reveal early pathogenic events and experimental work provides conflicting conclusions. The inconsistencies in recapitulating toxicity observed in ALS *C9ORF72* gain‐of‐function models could be because of the: (a) methodological difficulties in cloning and expressing GC‐rich repeats at a length that is observed in patients leading to inconsistent mimicking of repeat pathology; (b) over‐expression of repeat expansions in different in vitro and in vivo models, which may not necessarily mimic the endogenous levels of the expanded *C9ORF72‐S* or *‐AS* transcripts observed in patients; and (c) sequestration of a different set of RBPs by RNA foci in humans and different experimental models (Balendra & Isaacs, 2018).

Targeting of *C9ORF72‐S* by antisense oligonucleotides (ASOs) and of the G‐quadruplex structure formed by *C9ORF72‐S* by specific binding molecules have been explored as therapeutic strategies for C9‐ALS (Donnelly et al., 2013; Jiang, Zhu, et al., 2016; Lagier‐Tourenne et al., 2013; Simone et al., 2018; Zamiri et al., 2014; Zhang et al., 2015). ASOs were effective in reducing RNA foci and reversed gene expression changes in C9‐ALS hiPSC‐derived MNs (Sareen et al., 2013). Even specific targeting of the repeat expansion by ASOs mitigated pathological hallmarks of ALS. In addition to reducing intranuclear accumulation of *C9ORF72‐S* in RNA foci and normalizing dysregulated gene expression, ASOs were effective in reducing sequestration of the RBP adenosine deaminase RNA‐specific B2 (inactive) to the repeat expansion and in reversing glutamate toxicity in C9‐ALS patient fibroblasts and neurons (Donnelly et al., 2013). However, ASOs targeting the sense strand were not effective in reducing the abundant RNA foci formed by *C9ORF72‐AS* (Lagier‐Tourenne et al., 2013). This could limit clinical efficacy if toxicity in patients is mainly caused by *C9ORF72‐AS* RNA. Another important consideration for ASO therapy are off‐target effects. The application of ASOs could lead to adverse side effects, because of their off‐target effects depending on the sequence and modification chemistry of the motifs used to stabilize the ASO‐molecule for protection against cellular degradation (Schoch & Miller, 2017). Off‐target effects of ASOs are caused by binding to other mRNAs leading to an undesirable biological effect and activation of the immune system. Non‐specific target binding is avoided by careful selection of candidate ASOs. Human clinical ASO candidates are routinely screened in silico for potential mismatches and tested empirically for their effect on mRNAs (Kamola et al., 2015; Monia et al., 1992). Activation of the immune system is because of the recognition of ASOs with certain chemical modifications as foreign DNA by toll‐like receptors. It is reported that, unmethylated cytosine‐phosphorus‐guanine motifs in the ASO are recognized by toll‐like receptors‐9, resulting in antibody production, activation of T lymphocytes, natural killer cells and release of cytokines, interleukins (IL)‐6, IL‐12 and interferon (INF)‐γ (Agrawal & Kandimalla, 2004; Karaki et al., 2019; Vollmer et al., 2004). However, adaptations in the ASO design have largely mitigated this effect (Bennett & Swayze, 2010; Scoles et al., 2019; Sewell et al., 2002). Despite extensive research in ASO development, unrecognized off‐target issues remain (Schoch & Miller, 2017). So, it is important that each ASO molecule is evaluated independently and considered for a complete toxicological assessment.

Different effects of antisense transcripts on their sense counterparts have been described (Kanduri, 2008; Khorkova et al., 2014; Malecová & Morris, 2010; Modarresi et al., 2012; Yu et al., 2008). First, formation of double‐stranded RNA structures by hybridization of overlapping complementary RNA sequences in sense and antisense transcripts can activate the RNA interference pathway (Polikepahad & Corry, 2013; Werner et al., 2014). Second, antisense transcripts may cause transcriptional interference via the displacement of sense transcription factors in the sense promoter region (Wight & Werner, 2013). Third, antisense transcripts may epigenetically regulate sense transcripts through recruitment of chromatin remodelling factors and modulation of their activity (Kanduri, 2008; Malecová & Morris, 2010; Yu et al., 2008). Given these possible roles of antisense transcripts, sense oligonucleotides (SOs) designed against antisense transcripts may be a valuable therapeutic strategy. However, further studies are needed to better define the role of *C9ORF72‐AS* in C9‐ALS.

### 3.4 *ATAXIN*

The ALS risk gene *ataxin‐2* (*ATXN2*) also gives rise to antisense transcripts, *ATXN2‐AS*, with increased expression in ALS patient tissue (Li, Sun, et al., 2016). ATXN2 protein is ubiquitously expressed in neuronal and non‐neuronal tissues and is localized to the cytoplasm (Huynh et al., 1999). ATXN2 associates with polyribosomes in the endoplasmic reticulum and regulates mRNA translation (Satterfield & Pallanck, 2006). Further, it influences mRNA maturation, energy metabolism and endocytosis (Huynh et al., 1999; Magaña et al., 2013). In ALS, mutant ATXN2 interacts with FUS and TDP‐43 in an RNA‐dependent interaction manner which contributes to disease pathogenesis (Elden et al., 2010; Van den Heuvel et al., 2014; Yokoshi et al., 2014). The interaction of ATXN2 and TDP‐43 was first identified in a screen for toxic modifiers of TDP‐43. This work showed that an increase in the length of a polyQ repeat in ATXN2 (from 22 normal to 27–33 glutamines in ALS) is significantly associated with enhanced risk for developing ALS (Elden et al., 2010). ATXN2 polyQ repeat expansions of more than 33 repeats are associated with another neurodegenerative disease spinocerebellar ataxia type 2 (SCA2; Li, Sun, et al., 2016; Van den Heuvel et al., 2014). SCA2 is an autosomal‐dominant disorder caused by the expansion of CAG repeats in the N‐terminal coding region of *ATXN2*. Clinical features of SCA2 are characterized mainly by limb and gait ataxia, dysarthria and abnormal eye movements (Cancel et al., 1997; Geschwind et al., 1997). The symptoms of SCA2 are found to be clearly of cerebellar origin and involve cerebellar circuits. However, a typical ocular‐dependent feature is also observed in SCA2, where slow or absent saccades are displayed arising from the degeneration of oculomotor neurons. Other frequently observed symptoms include dystonia, myoclonus as well as neuropathy, muscle spasticity and frontal‐executive dysfunction (Geschwind et al., 1997; Scoles & Pulst, 2018). The *ATXN2* locus is bidirectionally transcribed in both ALS and SCA2 patients carrying an *ATXN2* repeat expansion. In three human ALS lymphoblastoid lines a non‐translatable *ATXN2‐AS* transcript was detected from both the normal and expanded allele (with intermediate CAG expansions [31–32 triplets]). Toxicity assays were performed to confirm the toxic nature of the expanded *ATXN2‐AS* transcript in SK‐N‐MC neuroblastoma cells (Li, Sun, et al., 2016). These mutant CAG/CUG transcripts aberrantly interact with RBPs involved in, for example, ribosomal RNA (rRNA) processing and splicing (Figure 1c; Swinnen et al., 2020). Despite these promising results, whether and if so how *ATXN2‐AS* contributes to ALS pathogenesis remains to be established.

### 3.5 Other lncRNAs in ALS

Whole transcriptome RNA sequencing (RNA‐seq) analysis in peripheral blood mononuclear cells (PBMCs) and spinal cord from sALS and fALS patients identified several differentially expressed lncRNAs (Gagliardi, et al., 2018). Several of these lncRNAs were antisense RNAs that were deregulated in sALS, for example, *ZEB1‐AS* and *ZBTB11‐AS*, and with sense counterparts that have reported functions in transcription regulation (Keightley et al., 2017; Li, Xie, et al., 2016). Interestingly, a few of these antisense lncRNAs can potentially target genes associated with neurodegenerative diseases, that is, *UBXN7‐AS*, *ATG10‐AS* and *ADORA2A‐AS* (Guo & Qi, 2017; Lee et al., 2015; Villar‐Menéndez et al., 2014). The detection of lncRNAs in PBMCs and blood raises the possibility that lncRNAs may be used as biomarkers for assessing clinical disease course or characteristics. Intriguingly, several of the lncRNAs detected in healthy and diseased MNs have also been reported in blood, PBMCs or peripheral blood lymphocytes, for example, *NEAT1*, *MALAT1*, *MEG3* and *ZEB1‐AS1* (Fallah et al., 2019; Gagliardi, et al., 2018; Gao et al., 2016; Huang et al., 2017; Wang, Li, et al., 2019).

Other lncRNAs have been implicated in ALS (or FTD) through their link with proteins associated with ALS pathogenesis, for example, TDP‐43 or FUS. *MALAT1* showed increased expression and binding to TDP‐43 in cortical tissue of sporadic FTD patients, while *Meg3* was down‐regulated and showed decreased TDP‐43 binding (Tollervey et al., 2011; Zhang et al., 2017). TDP‐43 can associate with several other lncRNAs, including *BDNFOS* and *TFEBα* (in SHSY5Y cells) and *Myolinc* (in muscle cells; Militello et al., 2018; Xiao et al., 2011). Expression of the lncRNA *heat‐shock rna ω (hsrω)* is positively regulated by TDP‐43, with direct binding of TDP‐43 to the *hsrω* locus (Chung et al., 2018). The stress‐induced lncRNA *Satellite III repeat RNA (Sat III)* is the human orthologue of *Drosophila hsrω* and shows increased expression in human FTD patient tissue and in cellular models of TDP‐43 over‐expression (Chung et al., 2018).

The introduction of ALS‐associated mutations in *FUS* in mESC‐derived MNs affects the expression of several lncRNAs. A specific up‐regulation of the lncRNA *Lhx1os* and down‐regulation of *LncMN‐1 (2610316D01Rik)* and *LncMN‐2 (5330434G04Rik)* were observed in FUS^P517L/517L^ and FUS^−/−^ MNs, indicating that FUS loss‐of‐function affects lncRNA expression. *Lhx1os*, *LncMN‐1* and *LncMN‐2* are conserved between mouse and human, displaying increased expression during human in vitro MN differentiation (Biscarini et al., 2018). The *Drosophila* orthologue of human FUS, dFUS, interacts with *hsrω*. Depletion of *hsrω* leads to cytoplasmic mis‐localization and loss of nuclear dFUS function. Further, MN‐specific knockdown of *hsrω* impairs locomotion in larval and adult flies, and also induces anatomical defects in pre‐synaptic terminals of MNs (Lo Piccolo & Yamaguchi, 2017). Given these diverse interactions of TDP‐43 and FUS with lncRNAs it is likely that the cytoplasmic mis‐localization and perturbed functions of TDP‐43 and FUS in ALS MNs impacts on lncRNA distribution, expression and/or function that may contribute to MN degeneration and ALS.

### 3.6 Spinal muscular atrophy (SMA)

SMA is a neurodegenerative disease characterized by profound muscle weakness attributed to progressive spinal MN degeneration, observed within weeks to months after birth or even at birth. SMA is classified as a rare disease that has a worldwide incidence of one in 6,000 – 10,000 newborns. However, it is the second most common autosomal recessive disease after cystic fibrosis and is the most common monogenic defect leading to infant mortality (Lunn & Wang, 2016). SMA is caused by reduced levels of SMN protein because of recessive mutations of the *SMN1* gene and retention of a variable copy number of the highly homologous *SMN2* gene (Lefebvre et al., 1995; Lorson et al., 1999). The critical sequence difference between *SMN2* and *SMN1* is one nucleotide (C → T) in exon 7 at a splice enhancer site. This change results in alternative splicing of *SMN2* pre‐mRNA (Lorson et al., 1999; Monani et al., 1999). This leads to a preferential exclusion of exon 7 from most transcripts (*SMNΔ7*), leading to a truncated unstable SMN protein, contributing to reduced levels of overall functional SMN protein. Individuals affected by SMA show loss of SMN protein and carry at least one copy of *SMN2*. The number of *SMN2* copies inversely correlates with disease severity (Lefebvre et al., 1995). In addition to *SMN2* copy number, other modifiers are known to modulate SMA clinical severity (Crawford et al., 2012). Therapy development for SMA has focused on increasing SMN expression and, as outlined below, lncRNAs may be valuable targets for modulating SMN expression. A lncRNA that arises from the antisense strand of SMN, *SMN‐AS1*, represses SMN expression (d’Ydewalle et al., 2017; Woo et al., 2017). Similar to what is described for *C9ORF72‐AS* and *ATXN2‐AS*, *SMN‐AS1* is a naturally occurring antisense transcript. *SMN1‐AS* mainly localizes to neural tissues and neurons, and its expression increases during neuronal differentiation, inversely correlating with SMN protein expression. Chromatin immunoprecipitation showed that *SMN1‐AS* mediates the recruitment of PRC2 to the *SMN2* promoter, suppressing SMN protein expression. This effect can be rescued by knockdown of *SMN1‐AS* or inhibition of PRC2 activity (d’Ydewalle et al., 2017; Woo et al., 2017). Targeting *SMN1‐AS* using ASOs together with SMN2 splice‐switching oligonucleotides increases SMN expression in a dose‐dependent manner and improves survival in a severe mouse model of SMA (d’Ydewalle et al., 2017). These studies provide an important proof‐of‐concept for targeting lncRNAs as a means of inducing SMN expression and modulating disease progression.

### 3.7 CircRNAs in motor neuron disease

Several studies have investigated circRNAs in the context of human disease (Chen, Li, et al., 2016). In recent years, transcriptome analyses have revealed that dysregulation of circRNAs is associated with complex neurological diseases such as Alzheimer's disease, PD, multiple system atrophy and ALS (Chen, Mills, et al., 2016; D’Ambra et al., 2019; Errichelli et al., 2017; Hanan et al., 2020; Huang et al., 2018; Wang et al., 2018). As outlined in previous sections, FUS is required for circRNA biogenesis (Errichelli et al., 2017). HiPSC‐derived MNs harbouring ALS‐linked FUS mutations (FUS‐P525L) also showed deregulation of specific circRNAs suggesting that these mutations affect circRNA biogenesis. CircRNAs may not only play a role in ALS pathogenesis but could also be exploited as therapeutic agents. A genome wide loss‐of‐function screen for modifiers of TDP‐43 toxicity identified (intronic) lariats as decoys for TDP‐43. Reduction of debranching enzyme 1 activity led to an increase in intronic lariats in this screen. The newly formed lariats acted as decoys and sequestered toxic cytoplasmic TDP‐43 aggregates, possibly preventing them from interacting with other RNAs and RBPs (Armakola et al., 2012). This study suggests the possibility of using circular lncRNAs (such as circRNAs) as decoys for toxic proteins in ALS. CircRNAs were also investigated as potential biomarkers in ALS using microarray analysis (Dolinar et al., 2019). Expression profiling of circRNAs in ALS was performed by comparing leukocyte samples from 12 patient and eight age‐ and sex‐matched healthy controls. Interestingly, several of the dysregulated circRNAs were located in gene loci that play key roles in ALS pathologies, for example, *hsa_circ_0001173* in vesicle‐associated membrane protein‐associated protein B/C (Nishimura et al., 2004). Three of the circRNA candidates (*hsa_circ_0023919*, *hsa_circ_0063411* and *hsa_circ_0088036*) showed similar differential expression patterns in a replication experiment as in the original microarray experiment. Where, *hsa_circ_0023919* was down‐regulated, *hsa_circ_0063411* and *hsa_circ_0088036* were up‐regulated in ALS patients, with good sensitivity and specificity for detection. This suggests that these candidate circRNAs could be used as diagnostic markers for sALS (Dolinar et al., 2019). While microarrays only allow detection of selected RNAs, RNAseq approaches can facilitate a more unbiased detection of circRNAs in the future, including of low abundant and novel transcripts, and in identification of their biomarker potential.

Overall, these studies hint at roles for circRNAs in the pathogenesis of ALS, and as diagnostic and therapeutic targets.

## 4 CONCLUSION

During the past years, a large body of experimental evidence has highlighted the role of lncRNAs in MN development. Up‐regulation of the expression of linear and circular lncRNAs during MN development, conservation of these RNA molecules across species and their involvement in polycomb repressive complex 1/2 recruitment and function are common observations. Interestingly, often the same cluster of lncRNAs that is regulated during MN development is dysregulated in a MND context (e.g. *Lhx1os*, *lncMN‐1*, *lncMN‐2*). These results suggest that lncRNAs are essential for normal MN development and that their dysregulation could underlie the pathogenesis of diseases such as ALS and SMA. The main challenge in the field is to identify ‘key’ downstream targets that are affected by lncRNA dysregulation and that lead to MN degeneration. Future work should focus on downstream targets that go beyond the direct interactors of lncRNAs but that instead unveil underlying molecular networks. For this, development of both genetic tools and innovative in vitro/in vivo models is essential. Taking advantage of iPSC technology and genome editing for generating patient‐derived models (and isogenic controls) will be the key for dissecting the different roles of lncRNAs. Knockdown approaches, co‐immunoprecipitation and argonaute 2‐CLIP experiments combined with proteomics and next‐generation sequencing analysis of lncRNAs are part of the toolbox that could reveal the nature and consequences of lncRNA interactions. Such experimental strategies would allow the establishment of a direct link between lncRNA‐related targets and functions and consequent phenotypic alterations in MNs. Such improved understanding of the mechanism‐of‐action and roles of lncRNAs will open up exciting ideas for molecular strategies for treating MNDs. An interesting hypothesis that deserves further investigation is the idea that lncRNAs may contribute to the selective vulnerability of specific subtypes of MNs. Other classes of ncRNAs, including miRNAs, can mediate the subtype‐specific resistance of MNs to degenerate (Hoye et al., 2017; Tung et al., 2019). For example, reduced expression of the *miR‐17–92* cluster of miRNAs in limb innervating lateral motor column‐motor neurons (LMC‐MNs) is associated with increased vulnerability of LMC‐MNs to degenerate (Kanning et al., 2010; Tung et al., 2019). LncRNAs may subserve similar functions, especially given their ability to influence miRNA function. Sex‐specific expression of lncRNAs also has been reported and it is interesting from a disease perspective as MNDs such as ALS and X‐linked SMA occur more frequently in males as compared to females (Dressman et al., 2007; Wijesekera & Leigh, 2009). Currently, not much is known about potential sex‐specific expression of the lncRNAs but establishing such expression patterns would be the first step to probe sex‐specific lncRNA functions in healthy and diseased MNs.

Overall, accumulating experimental evidence hints at important roles for lncRNAs in MN development and disease. Although in many instances further work is needed to dissect how lncRNAs function in developing MNs and why lncRNA dysregulation contributes to MNDs, it is clear that such knowledge could aid the development of diagnostic and therapeutic approaches for treating MN pathologies like ALS and SMA.

## CONFLICT OF INTEREST

The authors declare that there is no conflict of interest regarding the publication of this article.

## References

[jnc15198-bib-0001] Agrawal, S. , & Kandimalla, E. R. (2004). Antisense and siRNA as agonists of Toll‐like receptors. Nature Biotechnology, 22(12), 1533–1537. 10.1038/nbt1042 15583662

[jnc15198-bib-0002] Albertson, D. N. , Schmidt, C. J. , Kapatos, G. , & Bannon, M. J. (2006). Distinctive profiles of gene expression in the human nucleus accumbens associated with cocaine and heroin abuse. Neuropsychopharmacology, 31, 2304–2312. 10.1038/sj.npp.1301089 16710320 PMC2239258

[jnc15198-bib-0003] Al‐Chalabi, A. , & Hardiman, O. (2013). The epidemiology of ALS: A conspiracy of genes, environment and time. Nature Reviews. Neurology, 9, 617–628. 10.1038/nrneurol.2013.203 24126629

[jnc15198-bib-0004] Al‐Chalabi, A. , Hardiman, O. , Kiernan, M. C. , Chiò, A. , Rix‐Brooks, B. , & van den Berg, L. H. (2016). Amyotrophic lateral sclerosis: Moving towards a new classification system. The Lancet Neurology, 10.1016/S1474-4422(16)30199-5 27647646

[jnc15198-bib-0005] An, H. , Skelt, L. , Notaro, A. , Highley, J. R. , Fox, A. H. , La Bella, V. , Buchman, V. L. , & Shelkovnikova, T. A. (2019). ALS‐linked FUS mutations confer loss and gain of function in the nucleus by promoting excessive formation of dysfunctional paraspeckles. Acta Neuropathologica Communications, 7, 7. 10.1186/s40478-019-0658-x 30642400 PMC6330737

[jnc15198-bib-0006] Armakola, M. , Higgins, M. J. , Figley, M. D. , Barmada, S. J. , Scarborough, E. A. , Diaz, Z. , Fang, X. , Shorter, J. , Krogan, N. J. , Finkbeiner, S. , Farese, R. V. , & Gitler, A. D. (2012). Inhibition of RNA lariat debranching enzyme suppresses TDP‐43 toxicity in ALS disease models. Nature Genetics, 44, 1302–1309. 10.1038/ng.2434 23104007 PMC3510335

[jnc15198-bib-0007] Assi, H. A. , Garavís, M. , González, C. , & Damha, M. J. (2018). I‐motif DNA: Structural features and significance to cell biology. Nucleic Acids Research, 10.1093/nar/gky735 PMC6144788 30124962

[jnc15198-bib-0008] Balendra, R. , & Isaacs, A. M. (2018). C9orf72‐mediated ALS and FTD: Multiple pathways to disease. Nature Reviews Neurology, 14, 544–558. 10.1038/s41582-018-0047-2 30120348 PMC6417666

[jnc15198-bib-0009] Banerjee, A. , Vest, K. E. , Pavlath, G. K. , & Corbett, A. H. (2017). Nuclear poly(A) binding protein 1 (PABPN1) and matrin3 interact in muscle cells and regulate RNA processing. Nucleic Acids Research, 45, 10706–10725. 10.1093/nar/gkx786 28977530 PMC5737383

[jnc15198-bib-0010] Barry, G. , Briggs, J. A. , Vanichkina, D. P. , Poth, E. M. , Beveridge, N. J. , Ratnu, V. S. , Nayler, S. P. , Nones, K. , Hu, J. , Bredy, T. W. , Nakagawa, S. , Rigo, F. , Taft, R. J. , Cairns, M. J. , Blackshaw, S. , Wolvetang, E. J. , & Mattick, J. S. (2014). The long non‐coding RNA Gomafu is acutely regulated in response to neuronal activation and involved in schizophrenia‐associated alternative splicing. Molecular Psychiatry, 19, 486–494. 10.1038/mp.2013.45 23628989

[jnc15198-bib-0011] Basu, S. , Müller, F. , & Sanges, R. (2013). Examples of sequence conservation analyses capture a subset of mouse long non‐coding RNAs sharing homology with fish conserved genomic elements. BMC Bioinformatics, 14, S14. 10.1186/1471-2105-14-S7-S14 PMC3633045 23815359

[jnc15198-bib-0012] Bennett, C. F. , & Swayze, E. E. (2010). RNA targeting therapeutics: Molecular mechanisms of antisense oligonucleotides as a therapeutic platform. Annual Review of Pharmacology and Toxicology, 50, 259–293. 10.1146/annurev.pharmtox.010909.105654 20055705

[jnc15198-bib-0013] Biscarini, S. , Capauto, D. , Peruzzi, G. , Lu, L. , Colantoni, A. , Santini, T. , Shneider, N. A. , Caffarelli, E. , Laneve, P. , & Bozzoni, I. (2018). Characterization of the lncRNA transcriptome in mESC‐derived motor neurons: Implications for FUS‐ALS. Stem Cell Res., 27, 172–179. 10.1016/j.scr.2018.01.037 29449089

[jnc15198-bib-0014] Blokhuis, A. M. , Groen, E. J. N. , Koppers, M. , Van Den Berg, L. H. , & Pasterkamp, R. J. (2013). Protein aggregation in amyotrophic lateral sclerosis. Acta Neuropathologica, 10.1007/s00401-013-1125-6 PMC3661910 23673820

[jnc15198-bib-0015] Bonnefond, A. , Vaillant, E. , Philippe, J. , Skrobek, B. , Lobbens, S. , Yengo, L. , Huyvaert, M. , Cavé, H. , Busiah, K. , Scharfmann, R. , Polak, M. , Abdul‐Rasoul, M. , Froguel, P. , & Vaxillaire, M. (2013). Transcription factor gene MNX1 is a novel cause of permanent neonatal diabetes in a consanguineous family. Diabetes Metab., 39, 276–280. 10.1016/j.diabet.2013.02.007 23562494

[jnc15198-bib-0016] Braconi, C. , Kogure, T. , Valeri, N. , Huang, N. , Nuovo, G. , Costinean, S. , Negrini, M. , Miotto, E. , Croce, C. M. , & Patel, T. (2011). MicroRNA‐29 can regulate expression of the long non‐coding RNA gene MEG3 in hepatocellular cancer. Oncogene, 30, 4750–4756. 10.1038/onc.2011.193 21625215 PMC4292930

[jnc15198-bib-0017] Briese, M. , Saal, L. , Appenzeller, S. , Moradi, M. , Baluapuri, A. , & Sendtner, M. (2016). Whole transcriptome profiling reveals the RNA content of motor axons. Nucleic Acids Research, 44, e33. 10.1093/nar/gkv1027 26464439 PMC4770199

[jnc15198-bib-0018] Burguete, A. S. , Almeida, S. , Gao, F.‐B. , Kalb, R. , Akins, M. R. , & Bonini, N. M. (2015). GGGGCC microsatellite RNA is neuritically localized, induces branching defects, and perturbs transport granule function. Elife, 4, 10.7554/eLife.08881 PMC4758954 26650351

[jnc15198-bib-0019] Burk, K. , & Pasterkamp, R. J. (2019). Disrupted neuronal trafficking in amyotrophic lateral sclerosis. Acta Neuropathologica, 137, 859–877. 10.1007/s00401-019-01964-7 30721407 PMC6531423

[jnc15198-bib-0020] Burrell, J. R. , Halliday, G. M. , Kril, J. J. , Ittner, L. M. , Götz, J. , Kiernan, M. C. , & Hodges, J. R. (2016). The frontotemporal dementia‐motor neuron disease continuum. Lancet, 10.1016/S0140-6736(16)00737-6 26987909

[jnc15198-bib-0021] Cabili, M. , Trapnell, C. , Goff, L. , Koziol, M. , Tazon‐Vega, B. , Regev, A. , & Rinn, J. L. (2011). Integrative annotation of human large intergenic noncoding RNAs reveals global properties and specific subclasses. Genes and Development, 25, 1915–1927. 10.1101/gad.17446611 21890647 PMC3185964

[jnc15198-bib-0022] Cancel, G. , Dürr, A. , Didierjean, O. , Imbert, G. , Bürk, K. , Lezin, A. , Belal, S. , Benomar, A. , Abada‐Bendib, M. , Vial, C. , Guimarães, J. , Chneiweiss, H. , Stevanin, G. , Yvert, G. , Abbas, N. , Saudou, F. , Lebre, A. S. , Yahyaoui, M. , Hentati, F. , … Brice, A. (1997). Molecular and clinical correlations in spinocerebellar ataxia 2: A study of 32 families. Human Molecular Genetics, 6, 709–715. 10.1093/hmg/6.5.709 9158145

[jnc15198-bib-0023] Carninci, P. , Kasukawa, T. , Katayama, S. , Gough, J. , Frith, M. C. , Maeda, N. , Oyama, R. , & Ravasi, T. (2005). Transcriptional landscape of the Mammalian Genome. Science, 80‐. ). 309, 1559–1563. 10.1126/science.1112014 16141072

[jnc15198-bib-0024] Chaudhry, M. A. (2014). Small nucleolar RNA host genes and long non‐coding RNA responses in directly irradiated and bystander cells. Cancer Biotherapy and Radiopharmaceuticals, 29, 135–141. 10.1089/cbr.2013.1574 24502193

[jnc15198-bib-0025] Chaytow, H. , Huang, Y. T. , Gillingwater, T. H. , & Faller, K. M. E. (2018). The role of survival motor neuron protein (SMN) in protein homeostasis. Cellular and Molecular Life Sciences, 75, 3877–3894. 10.1007/s00018-018-2849-1 29872871 PMC6182345

[jnc15198-bib-0026] Chen, B. J. , Mills, J. D. , Takenaka, K. , Bliim, N. , Halliday, G. M. , & Janitz, M. (2016). Characterization of circular RNAs landscape in multiple system atrophy brain. Journal of Neurochemistry, 139, 485–496. 10.1111/jnc.13752 27470294

[jnc15198-bib-0027] Chen, K. W. , & Chen, J. A. (2020). Functional roles of long non‐coding RNAs in motor neuron development and disease. Journal of Biomedical Science, 27, 1–14. 10.1186/s12929-020-00628-z 32093746 PMC7041250

[jnc15198-bib-0028] Chen, L. L. (2016). The biogenesis and emerging roles of circular RNAs. Nature Reviews Molecular Cell Biology, 17, 205–211. 10.1038/nrm.2015.32 26908011

[jnc15198-bib-0029] Chen, L. L. , & Carmichael, G. G. (2009). Altered nuclear retention of mRNAs containing inverted repeats in human embryonic stem cells: Functional role of a nuclear noncoding RNA. Molecular Cell, 35, 467–478. 10.1016/j.molcel.2009.06.027 19716791 PMC2749223

[jnc15198-bib-0030] Chen, W. , & Schuman, E. (2016). Circular RNAs in brain and other tissues: A functional enigma. Trends in Neurosciences, 39, 597–604. 10.1016/j.tins.2016.06.006 27445124

[jnc15198-bib-0031] Chen, Y. , Li, C. , Tan, C. , & Liu, X. (2016). Circular RNAs: A new frontier in the study of human diseases. Journal of Medical Genetics, 10.1136/jmedgenet-2016-103758 26945092

[jnc15198-bib-0032] Cheng, W. , Wang, S. , Zhang, Z. , Morgens, D. W. , Hayes, L. R. , Lee, S. , Portz, B. , Xie, Y. , Nguyen, B. V. , Haney, M. S. , Yan, S. , Dong, D. , Coyne, A. N. , Yang, J. , Xian, F. , Cleveland, D. W. , Qiu, Z. , Rothstein, J. D. , Shorter, J. , … Sun, S. (2019). CRISPR‐Cas9 screens identify the RNA helicase DDX3X as a repressor of C9ORF72 (GGGGCC)n repeat‐associated non‐AUG translation. Neuron, 104, 885–898.e8. 10.1016/j.neuron.2019.09.003 31587919 PMC6895427

[jnc15198-bib-0033] Chew, J. , Gendron, T. F. , Prudencio, M. , Sasaguri, H. , Zhang, Y. J. , Castanedes‐Casey, M. , Lee, C. W. , Jansen‐West, K. , Kurti, A. , Murray, M. E. , Bieniek, K. F. , Bauer, P. O. , Whitelaw, E. C. , Rousseau, L. , Stankowski, J. N. , Stetler, C. , Daughrity, L. M. , Perkerson, E. A. , Desaro, P. , … Petrucelli, L. (2015). C9ORF72 repeat expansions in mice cause TDP‐43 pathology, neuronal loss, and behavioral deficits. Science, 348, 1151–1154. 10.1126/science.aaa9344 25977373 PMC4692360

[jnc15198-bib-0034] Chodroff, R. A. , Goodstadt, L. , Sirey, T. M. , Oliver, P. L. , Davies, K. E. , Green, E. D. , Molnár, Z. , & Ponting, C. P. (2010). Long noncoding RNA genes: Conservation of sequence and brain expression among diverse amniotes. Genome Biology, 11, R72. 10.1186/gb-2010-11-7-r72 20624288 PMC2926783

[jnc15198-bib-0035] Chujo, T. , Yamazaki, T. , & Hirose, T. (2016). Architectural RNAs (arcRNAs): A class of long noncoding RNAs that function as the scaffold of nuclear bodies. Biochimica Et Biophysica Acta (BBA) ‐ Gene Regulatory Mechanisms, 1859(1), 139–146. 10.1016/j.bbagrm.2015.05.007 26021608

[jnc15198-bib-0036] Chujo, T. , Yamazaki, T. , Kawaguchi, T. , Kurosaka, S. , Takumi, T. , Nakagawa, S. , & Hirose, T. (2017). Unusual semi‐extractability as a hallmark of nuclear body‐associated architectural noncoding RNA s. EMBO Journal, 36, 1447–1462. 10.15252/embj.201695848 PMC5430218 28404604

[jnc15198-bib-0037] Chung, C.‐Y. , Berson, A. , Kennerdell, J. R. , Sartoris, A. , Unger, T. , Porta, S. , Kim, H.‐J. , Smith, E. R. , Shilatifard, A. , Van Deerlin, V. , Lee, V. M. Y. , Chen‐Plotkin, A. , & Bonini, N. M. (2018). Aberrant activation of non‐coding RNA targets of transcriptional elongation complexes contributes to TDP‐43 toxicity. Nature Communications, 9, 4406. 10.1038/s41467-018-06543-0 PMC6199344 30353006

[jnc15198-bib-0038] Clemson, C. M. , Hutchinson, J. N. , Sara, S. A. , Ensminger, A. W. , Fox, A. H. , Chess, A. , & Lawrence, J. B. (2009). An Architectural role for a nuclear noncoding RNA: NEAT1 RNA is essential for the structure of paraspeckles. Molecular Cell, 33, 717–726. 10.1016/j.molcel.2009.01.026 19217333 PMC2696186

[jnc15198-bib-0039] Cooper‐Knock, J. , Higginbottom, A. , Stopford, M. J. , Highley, J. R. , Ince, P. G. , Wharton, S. B. , Pickering‐Brown, S. , Kirby, J. , Hautbergue, G. M. , & Shaw, P. J. (2015). Antisense RNA foci in the motor neurons of C9ORF72‐ALS patients are associated with TDP‐43 proteinopathy. Acta Neuropathologica, 130, 63–75. 10.1007/s00401-015-1429-9 25943887 PMC4468790

[jnc15198-bib-0040] Crawford, T. O. , Paushkin, S. V. , Kobayashi, D. T. , Forrest, S. J. , Joyce, C. L. , Finkel, R. S. , Kaufmann, P. , Swoboda, K. J. , Tiziano, D. , Lomastro, R. , Li, R. H. , Trachtenberg, F. L. , Plasterer, T. , & Chen, K. S. (2012). Evaluation of SMN protein, transcript, and copy number in the biomarkers for spinal muscular atrophy (BforSMA) clinical study. PLoS One, 7, e33572. 10.1371/journal.pone.0033572 22558076 PMC3338744

[jnc15198-bib-0041] D’Ambra, E. , Capauto, D. , & Morlando, M. (2019). Exploring the regulatory role of circular RNAs in neurodegenerative disorders. International Journal of Molecular Sciences, 20, 5477. 10.3390/ijms20215477 PMC6862314 31689888

[jnc15198-bib-0042] d’Ydewalle, C. , Ramos, D. M. , Pyles, N. J. , Ng, S. Y. , Gorz, M. , Pilato, C. M. , Ling, K. , Kong, L. , Ward, A. J. , Rubin, L. L. , Rigo, F. , Bennett, C. F. , & Sumner, C. J. (2017). The antisense transcript SMN‐AS1 regulates SMN expression and is a novel therapeutic target for spinal muscular atrophy. Neuron, 93, 66–79. 10.1016/j.neuron.2016.11.033 28017471 PMC5223741

[jnc15198-bib-0043] Davis, I. J. , Hsi, B. L. , Arroyo, J. D. , Vargas, S. O. , Yeh, Y. A. , Motyckova, G. , Valencia, P. , Perez‐Atayde, A. R. , Argani, P. , Ladanyi, M. , Fletcher, J. A. , & Fisher, D. E. (2003). Cloning of an Alpha‐TFEB fusion in renal tumors harboring the t(6;11)(p21;q13) chromosome translocation. Proceedings of the National Academy of Sciences of the United States of America, 100, 6051–6056. 10.1073/pnas.0931430100 12719541 PMC156324

[jnc15198-bib-0044] DeJesus‐Hernandez, M. , Finch, N. C. A. , Wang, X. , Gendron, T. F. , Bieniek, K. F. , Heckman, M. G. , Vasilevich, A. , Murray, M. E. , Rousseau, L. , Weesner, R. , Lucido, A. , Parsons, M. , Chew, J. , Josephs, K. A. , Parisi, J. E. , Knopman, D. S. , Petersen, R. C. , Boeve, B. F. , Graff‐Radford, N. R. , … Rademakers, R. (2017). In‐depth clinico‐pathological examination of RNA foci in a large cohort of C9ORF72 expansion carriers. Acta Neuropathologica, 134, 255–269. 10.1007/s00401-017-1725-7 28508101 PMC5508036

[jnc15198-bib-0045] DeJesus‐Hernandez, M. , Mackenzie, I. R. , Boeve, B. F. , Boxer, A. L. , Baker, M. , Rutherford, N. J. , Nicholson, A. M. , Finch, N. C. A. , Flynn, H. , Adamson, J. , Kouri, N. , Wojtas, A. , Sengdy, P. , Hsiung, G. Y. R. , Karydas, A. , Seeley, W. W. , Josephs, K. A. , Coppola, G. , Geschwind, D. H. , … Rademakers, R. (2011). Expanded GGGGCC hexanucleotide repeat in noncoding region of C9ORF72 causes chromosome 9p‐linked FTD and ALS. Neuron, 72, 245–256. 10.1016/j.neuron.2011.09.011 21944778 PMC3202986

[jnc15198-bib-0046] Derrien, T. , Johnson, R. , Bussotti, G. , Tanzer, A. , Djebali, S. , Tilgner, H. , Guernec, G. , Martin, D. , Merkel, A. , Knowles, D. G. , Lagarde, J. , Veeravalli, L. , Ruan, X. , Ruan, Y. , Lassmann, T. , Carninci, P. , Brown, J. B. , Lipovich, L. , Gonzalez, J. M. , … Guigó, R. (2012). The GENCODE v7 catalog of human long noncoding RNAs: Analysis of their gene structure, evolution, and expression. Genome Research, 22, 1775–1789. 10.1101/gr.132159.111 22955988 PMC3431493

[jnc15198-bib-0047] Dinger, M. E. , Amara, P. P. , Mercer, T. R. , Pang, K. C. , Bruce, S. J. , Gardiner, B. B. , Askarian‐Amiri, M. E. , Ru, K. , Soldà, G. , Simons, C. , Sunkin, S. M. , Crowe, M. L. , Grimmond, S. M. , Perkins, A. C. , & Mattick, J. S. (2008). Long noncoding RNAs in mouse embryonic stem cell pluripotency and differentiation. Genome Research, 18, 1433–1445. 10.1101/gr.078378.108 18562676 PMC2527704

[jnc15198-bib-0048] Djebali, S. , Davis, C. A. , Merkel, A. , Dobin, A. , Lassmann, T. , Mortazavi, A. , Tanzer, A. , Lagarde, J. , Lin, W. , Schlesinger, F. , Xue, C. , Marinov, G. K. , Khatun, J. , Williams, B. A. , Zaleski, C. , Rozowsky, J. , Röder, M. , Kokocinski, F. , Abdelhamid, R. F. , … Gingeras, T. R. (2012). Landscape of transcription in human cells. Nature, 489, 101–108. 10.1038/nature11233 22955620 PMC3684276

[jnc15198-bib-0049] Dodd, D. W. , Tomchick, D. R. , Corey, D. R. , & Gagnon, K. T. (2016). Pathogenic C9ORF72 antisense repeat RNA forms a double helix with tandem C: C mismatches. Biochemistry, 55, 1283–1286. 10.1021/acs.biochem.6b00136 26878348

[jnc15198-bib-0050] Dolinar, A. , Koritnik, B. , Glavač, D. , & Ravnik‐Glavač, M. (2019). Circular RNAs as potential blood biomarkers in amyotrophic lateral sclerosis. Molecular Neurobiology, 56, 8052–8062. 10.1007/s12035-019-1627-x 31175544 PMC6834740

[jnc15198-bib-0051] Donnelly, C. J. , Zhang, P. W. , Pham, J. T. , Heusler, A. R. , Mistry, N. A. , Vidensky, S. , Daley, E. L. , Poth, E. M. , Hoover, B. , Fines, D. M. , Maragakis, N. , Tienari, P. J. , Petrucelli, L. , Traynor, B. J. , Wang, J. , Rigo, F. , Bennett, C. F. , Blackshaw, S. , Sattler, R. , & Rothstein, J. D. (2013). RNA toxicity from the ALS/FTD C9ORF72 expansion is mitigated by antisense intervention. Neuron, 80, 415–428. 10.1016/j.neuron.2013.10.015 24139042 PMC4098943

[jnc15198-bib-0052] Douglas, A. G. L. (2018). Non‐coding RNA in C9orf72‐related amyotrophic lateral sclerosis and frontotemporal dementia: A perfect storm of dysfunction. Non‐coding RNA Research, 3, 178–187. 10.1016/j.ncrna.2018.09.001 30533567 PMC6260478

[jnc15198-bib-0053] Dressman, D. , Ahearn, M. E. , Yariz, K. O. , Basterrecha, H. , Martínez, F. , Palau, F. , Barmada, M. M. , Clark, R. D. , Meindl, A. , Wirth, B. , Hoffman, E. P. , & Baumbach‐Reardon, L. (2007). X‐linked infantile spinal muscular atrophy: Clinical definition and molecular mapping. Genetics in Medicine, 9, 52–60. 10.1097/GIM.0b013e31802d8353 17224690

[jnc15198-bib-0054] Dunham, I. , Kundaje, A. , Aldred, S. F. , Collins, P. J. , Davis, C. A. , Doyle, F. , Epstein, C. B. , Frietze, S. , Harrow, J. , Kaul, R. , Khatun, J. , Lajoie, B. R. , Landt, S. G. , Lee, B. K. , Pauli, F. , Rosenbloom, K. R. , Sabo, P. , Safi, A. , Sanyal, A. , … Lochovsky, L. (2012). An integrated encyclopedia of DNA elements in the human genome. Nature, 489, 57–74. 10.1038/nature11247 22955616 PMC3439153

[jnc15198-bib-0055] Ebbesen, K. K. , Kjems, J. , & Hansen, T. B. (2016). Circular RNAs: Identification, biogenesis and function. Biochimica Et Biophysica Acta (BBA)‐Gene Regulatory Mechanisms, 1859, 163–168. 10.1016/j.bbagrm.2015.07.007 26171810

[jnc15198-bib-0056] Elden, A. C. , Kim, H. J. , Hart, M. P. , Chen‐Plotkin, A. S. , Johnson, B. S. , Fang, X. , Armakola, M. , Geser, F. , Greene, R. , Lu, M. M. , Padmanabhan, A. , Clay‐Falcone, D. , McCluskey, L. , Elman, L. , Juhr, D. , Gruber, P. J. , Rüb, U. , Auburger, G. , Trojanowski, J. Q. , … Gitler, A. D. (2010). Ataxin‐2 intermediate‐length polyglutamine expansions are associated with increased risk for ALS. Nature, 466, 1069–1075. 10.1038/nature09320 20740007 PMC2965417

[jnc15198-bib-0057] Elling, R. , Chan, J. , & Fitzgerald, K. A. (2016). Emerging role of long noncoding RNAs as regulators of innate immune cell development and inflammatory gene expression. European Journal of Immunology, 46, 504–512. 10.1002/eji.201444558 26820238 PMC5404502

[jnc15198-bib-0058] Errichelli, L. , Dini Modigliani, S. , Laneve, P. , Colantoni, A. , Legnini, I. , Capauto, D. , Rosa, A. , De Santis, R. , Scarfò, R. , Peruzzi, G. , Lu, L. , Caffarelli, E. , Shneider, N. A. , Morlando, M. , & Bozzoni, I. (2017). FUS affects circular RNA expression in murine embryonic stem cell‐derived motor neurons. Nature Communications, 8, 1–11. 10.1038/ncomms14741 PMC5379105 28358055

[jnc15198-bib-0059] Fallah, H. , Azari, I. , Neishabouri, S. M. , Oskooei, V. K. , Taheri, M. , & Ghafouri‐Fard, S. (2019). Sex‐specific up‐regulation of lncRNAs in peripheral blood of patients with schizophrenia. Scientific Reports, 9, 1–8. 10.1038/s41598-019-49265-z 31484957 PMC6726592

[jnc15198-bib-0060] Fallini, C. , Bassell, G. J. , & Rossoll, W. (2012). Spinal muscular atrophy: The role of SMN in axonal mRNA regulation. Brain Research, 1462, 81–92. 10.1016/j.brainres.2012.01.044 22330725 PMC3360984

[jnc15198-bib-0061] Ferraiuolo, L. , Meyer, K. , Sherwood, T. W. , Vick, J. , Likhite, S. , Frakes, A. , Miranda, C. J. , Braun, L. , Heath, P. R. , Pineda, R. , Beattie, C. E. , Shaw, P. J. , Askwith, C. C. , McTigue, D. , & Kaspar, B. K. (2016). Oligodendrocytes contribute to motor neuron death in ALS via SOD1‐dependent mechanism. Proceedings of the National Academy of Sciences, 113, E6496–E6505. 10.1073/pnas.1607496113 PMC5081600 27688759

[jnc15198-bib-0062] Field, A. R. , Jacobs, F. M. J. , Fiddes, I. T. , Phillips, A. P. R. , Reyes‐Ortiz, A. M. , LaMontagne, E. , Whitehead, L. , Meng, V. , Rosenkrantz, J. L. , Olsen, M. , Hauessler, M. , Katzman, S. , Salama, S. R. , & Haussler, D. (2019). Structurally conserved primate LncRNAs are transiently expressed during human cortical differentiation and influence cell‐type‐specific genes. Stem Cell Reports, 12, 245–257. 10.1016/j.stemcr.2018.12.006 30639214 PMC6372947

[jnc15198-bib-0063] Fox, A. H. , & Lamond, A. I. (2010). Paraspeckles. Cold Spring Harbor Perspectives in Biology, 2, a000687. 10.1101/cshperspect.a000687 20573717 PMC2890200

[jnc15198-bib-0064] Fox, A. H. , Nakagawa, S. , Hirose, T. , & Bond, C. S. (2018). Paraspeckles: Where long noncoding RNA meets phase separation. Trends in Biochemical Sciences, 43(2), 124–135. 10.1016/j.tibs.2017.12.001 29289458

[jnc15198-bib-0065] Fratta, P. , Mizielinska, S. , Nicoll, A. J. , Zloh, M. , Fisher, E. M. C. , Parkinson, G. , & Isaacs, A. M. (2012). C9orf72 hexanucleotide repeat associated with amyotrophic lateral sclerosis and frontotemporal dementia forms RNA G‐quadruplexes. Scientific Reports, 2, 1016. 10.1038/srep01016 23264878 PMC3527825

[jnc15198-bib-0066] Freibaum, B. D. , Lu, Y. , Lopez‐Gonzalez, R. , Kim, N. C. , Almeida, S. , Lee, K. H. , Badders, N. , Valentine, M. , Miller, B. L. , Wong, P. C. , Petrucelli, L. , Kim, H. J. , Gao, F. B. , & Taylor, J. P. (2015). GGGGCC repeat expansion in C9orf72 compromises nucleocytoplasmic transport. Nature, 525, 129–133. 10.1038/nature14974 26308899 PMC4631399

[jnc15198-bib-0067] Freibaum, B. D. , & Taylor, J. P. (2017). The role of dipeptide repeats in C9ORF72‐related ALS‐FTD. Frontiers in Molecular Neuroscience, 10, 10.3389/fnmol.2017.00035 PMC5303742 28243191

[jnc15198-bib-0068] Gabanella, F. , Carissimi, C. , Usiello, A. , & Pellizzoni, L. (2005). The activity of the spinal muscular atrophy protein is regulated during development and cellular differentiation. Human Molecular Genetics, 14, 3629–3642. 10.1093/hmg/ddi390 16236758

[jnc15198-bib-0069] Gagliardi, S. , Pandini, C. , Garofalo, M. , Bordoni, M. , Pansarasa, O. , & Cereda, C. (2018). Long non coding RNAs and ALS: Still much to do. Non‐coding RNA Research, 3, 226–231. 10.1016/j.ncrna.2018.11.004 30533570 PMC6260474

[jnc15198-bib-0070] Gagliardi, S. , Zucca, S. , Pandini, C. , Diamanti, L. , Bordoni, M. , Sproviero, D. , Arigoni, M. , Olivero, M. , Pansarasa, O. , Ceroni, M. , Calogero, R. , & Cereda, C. (2018). Long non‐coding and coding RNAs characterization in peripheral blood mononuclear cells and spinal cord from amyotrophic lateral sclerosis patients. Scientific Reports, 8, 1–11. 10.1038/s41598-018-20679-5 29402919 PMC5799454

[jnc15198-bib-0071] Gao, C. , He, Z. , Li, J. , Li, X. , Bai, Q. , Zhang, Z. , Zhang, X. , Wang, S. , Xiao, X. , Wang, F. , Yan, Y. , Li, D. , Chen, L. , Zeng, X. , Xiao, Y. , Dong, G. , Zheng, Y. , Wang, Q. , & Chen, W. (2016). Specific long non‐coding RNAs response to occupational PAHs exposure in coke oven workers. Toxicology Reports, 3, 160–166. 10.1016/j.toxrep.2015.12.011 28959535 PMC5615781

[jnc15198-bib-0072] Gao, T. , Li, J. , Li, N. , Gao, Y. , Yu, L. , Zhuang, S. , Zhao, Y. , & Dong, X. (2020). Lncrps25 play an essential role in motor neuron development through controlling the expression of Olig2 in zebrafish. Journal of Cellular Physiology, 235, 3485–3496. 10.1002/jcp.29237 31549395 PMC13482657

[jnc15198-bib-0073] Gendron, T. F. , Bieniek, K. F. , Zhang, Y. J. , Jansen‐West, K. , Ash, P. E. A. , Caulfield, T. , Daughrity, L. , Dunmore, J. H. , Castanedes‐Casey, M. , Chew, J. , Cosio, D. M. , Van Blitterswijk, M. , Lee, W. C. , Rademakers, R. , Boylan, K. B. , Dickson, D. W. , & Petrucelli, L. (2013). Antisense transcripts of the expanded C9ORF72 hexanucleotide repeat form nuclear RNA foci and undergo repeat‐associated non‐ATG translation in c9FTD/ALS. Acta Neuropathologica, 126, 829–844. 10.1007/s00401-013-1192-8 24129584 PMC3830741

[jnc15198-bib-0074] Gendron, T. F. , Chew, J. , Stankowski, J. N. , Hayes, L. R. , Zhang, Y.‐J. , Prudencio, M. , Carlomagno, Y. , Daughrity, L. M. , Jansen‐West, K. , Perkerson, E. A. , O’Raw, A. , Cook, C. , Pregent, L. , Belzil, V. , van Blitterswijk, M. , Tabassian, L. J. , Lee, C. W. , Yue, M. , Tong, J. , … Poletti, L. (2017). Poly(GP) proteins are a useful pharmacodynamic marker for C9ORF72 ‐associated amyotrophic lateral sclerosis. Science Translational Medicine, 9, eaai7866. 10.1126/scitranslmed.aai7866 28356511 PMC5576451

[jnc15198-bib-0075] Geschwind, D. H. , Perlman, S. , Figueroa, C. P. , Treiman, L. J. , & Pulst, S. M. (1997). The prevalence and wide clinical spectrum of the spinocerebellar ataxia type 2 trinucleotide repeat in patients with autosomal dominant cerebellar ataxia. American Journal of Human Genetics, 60, 842–850. 9106530 PMC1712476

[jnc15198-bib-0076] Giavazzi, A. , Setola, V. , Simonati, A. , & Battaglia, G. (2006). Neuronal‐specific roles of the survival motor neuron protein: Evidence from survival motor neuron expression patterns in the developing human central nervous system. Journal of Neuropathology and Experimental Neurology, 10.1097/01.jnen.0000205144.54457.a3 16651888

[jnc15198-bib-0077] Groen, E. J. N. , Fumoto, K. , Blokhuis, A. M. , Engelen‐Lee, J. Y. , Zhou, Y. , van den Heuvel, D. M. A. , Koppers, M. , van Diggelen, F. , van Heest, J. , Demmers, J. A. A. , Kirby, J. , Shaw, P. J. , Aronica, E. , Spliet, W. G. M. , Veldink, J. H. , van den Berg, L. H. , & Pasterkamp, R. J. (2013). ALS‐associated mutations in FUS disrupt the axonal distribution and function of SMN. Human Molecular Genetics, 22, 3690–3704. 10.1093/hmg/ddt222 23681068

[jnc15198-bib-0078] Guo, X. , & Qi, X. (2017). VCP cooperates with UBXD1 to degrade mitochondrial outer membrane protein MCL1 in model of Huntington’s disease. Biochimica Et Biophysica Acta (BBA) ‐ Molecular Basis of Disease, 1863, 552–559. 10.1016/j.bbadis.2016.11.026 27913212 PMC5219860

[jnc15198-bib-0079] Guttman, M. , Amit, I. , Garber, M. , French, C. , Lin, M. F. , Feldser, D. , Huarte, M. , Zuk, O. , Carey, B. W. , Cassady, J. P. , Cabili, M. N. , Jaenisch, R. , Mikkelsen, T. S. , Jacks, T. , Hacohen, N. , Bernstein, B. E. , Kellis, M. , Regev, A. , Rinn, J. L. , & Lander, E. S. (2009). Chromatin signature reveals over a thousand highly conserved large non‐coding RNAs in mammals. Nature, 458, 223–227. 10.1038/nature07672 19182780 PMC2754849

[jnc15198-bib-0080] Haeusler, A. R. , Donnelly, C. J. , Periz, G. , Simko, E. A. J. , Shaw, P. G. , Kim, M.‐S. , Maragakis, N. J. , Troncoso, J. C. , Pandey, A. , Sattler, R. , Rothstein, J. D. , & Wang, J. (2014). C9orf72 nucleotide repeat structures initiate molecular cascades of disease. Nature, 507(7491), 195–200. 10.1038/nature13124 24598541 PMC4046618

[jnc15198-bib-0081] Hanan, M. , Simchovitz, A. , Yayon, N. , Vaknine, S. , Cohen‐Fultheim, R. , Karmon, M. , Madrer, N. , Rohrlich, T. M. , Maman, M. , Bennett, E. R. , Greenberg, D. S. , Meshorer, E. , Levanon, E. Y. , Soreq, H. , & Kadener, S. (2020). A Parkinson’s disease Circ RNA s Resource reveals a link between circ SLC 8A1 and oxidative stress. EMBO Molecular Medicine, 12, e11942. 10.15252/emmm.201911942. 32715657 PMC7507321

[jnc15198-bib-0082] He, D. , Wang, J. , Lu, Y. , Deng, Y. , Zhao, C. , Xu, L. , Chen, Y. , Hu, Y. C. , Zhou, W. , & Lu, Q. R. (2017). lncRNA functional networks in oligodendrocytes reveal stage‐specific myelination control by an lncOL1/Suz12 complex in the CNS. Neuron, 93, 362–378. 10.1016/j.neuron.2016.11.044 28041882 PMC5600615

[jnc15198-bib-0083] Hennig, S. , Kong, G. , Mannen, T. , Sadowska, A. , Kobelke, S. , Blythe, A. , Knott, G. J. , Iyer, S. S. , Ho, D. , Newcombe, E. A. , Hosoki, K. , Goshima, N. , Kawaguchi, T. , Hatters, D. , Trinkle‐Mulcahy, L. , Hirose, T. , Bond, C. S. , & Fox, A. H. (2015). Prion‐like domains in RNA binding proteins are essential for building subnuclear paraspeckles. Journal of Cell Biology, 210, 529–539. 10.1083/jcb.201504117 PMC4539981 26283796

[jnc15198-bib-0084] Hirose, T. , Virnicchi, G. , Tanigawa, A. , Naganuma, T. , Li, R. , Kimura, H. , Yokoi, T. , Nakagawa, S. , Bénard, M. , Fox, A. H. , & Pierron, G. (2014). NEAT1 long noncoding RNA regulates transcription via protein sequestration within subnuclear bodies. Molecular Biology of the Cell, 25, 169–183. 10.1091/mbc.E13-09-0558 24173718 PMC3873887

[jnc15198-bib-0085] Hoye, M. L. , Koval, E. D. , Wegener, A. J. , Hyman, T. S. , Yang, C. , O’Brien, D. R. , Miller, R. L. , Cole, T. , Schoch, K. M. , Shen, T. , Kunikata, T. , Richard, J. P. , Gutmann, D. H. , Maragakis, N. J. , Kordasiewicz, H. B. , Dougherty, J. D. , & Miller, T. M. (2017). MicroRNA profiling reveals marker of motor neuron disease in ALS models. Journal of Neuroscience, 37, 5574–5586. 10.1523/JNEUROSCI.3582-16.2017 28416596 PMC5452343

[jnc15198-bib-0086] Huang, J. L. , Qin, M. C. , Zhou, Y. , Xu, Z. H. , Yang, S. , Zhang, F. , Zhong, J. , Liang, M.‐K. , Chen, B. , Zhang, W.‐Y. , Wu, D.‐P. , & Zhong, Z.‐G. (2018). Comprehensive analysis of differentially expressed profiles of Alzheimer’s disease associated circular rnas in an Alzheimer’s disease mouse model. Aging (Albany, NY), 10, 253–265. 10.18632/aging.101387 29448241 PMC5842852

[jnc15198-bib-0087] Huang, S. , Qian, K. , Zhu, Y. , Huang, Z. , Luo, Q. , & Qing, C. (2017). Diagnostic value of the lncRNA NEAT1 in peripheral blood mononuclear cells of patients with sepsis. Disease Markers, 2017, 1–6. 10.1155/2017/7962836 PMC5804381 29463949

[jnc15198-bib-0088] Huynh, D. P. , Del Bigio, M. R. , Ho, D. H. , & Pulst, S. M. (1999). Expression of ataxin‐2 in brains from normal individuals and patients with Alzheimer’s disease and spinocerebellar ataxia 2. Annals of Neurology, 45, 232–241. 10.1002/1531-8249(199902)45:2<232:AID-ANA14>3.0.CO;2-7 9989626

[jnc15198-bib-0089] Jeck, W. R. , Sorrentino, J. A. , Wang, K. , Slevin, M. K. , Burd, C. E. , Liu, J. , Marzluff, W. F. , & Sharpless, N. E. (2013). Circular RNAs are abundant, conserved, and associated with ALU repeats. RNA, 19, 141–157. 10.1261/rna.035667.112 23249747 PMC3543092

[jnc15198-bib-0090] Jiang, C. , Li, Y. , Zhao, Z. , Lu, J. , Chen, H. , Ding, N. , Wang, G. , Xu, J. , & Li, X. (2016). Identifying and functionally characterizing tissue‐specific and ubiquitously expressed human lncRNAs. Oncotarget, 7, 7120–7133. 10.18632/oncotarget.6859 26760768 PMC4872773

[jnc15198-bib-0091] Jiang, J. , Zhu, Q. , Gendron, T. F. , Saberi, S. , McAlonis‐Downes, M. , Seelman, A. , Stauffer, J. E. , Jafar‐nejad, P. , Drenner, K. , Schulte, D. , Chun, S. , Sun, S. , Ling, S. C. , Myers, B. , Engelhardt, J. , Katz, M. , Baughn, M. , Platoshyn, O. , Marsala, M. , … Lagier‐Tourenne, C. (2016). Gain of toxicity from ALS/FTD‐linked repeat expansions in C9ORF72 is alleviated by antisense oligonucleotides targeting GGGGCC‐containing RNAs. Neuron, 90, 535–550. 10.1016/j.neuron.2016.04.006 27112497 PMC4860075

[jnc15198-bib-0092] Johnsson, P. , Lipovich, L. , Grandér, D. , & Morris, K. V. (2014). Evolutionary conservation of long non‐coding RNAs; sequence, structure, function. Biochimica Et Biophysica Acta (BBA) ‐ General Subjects, 1840(3), 1063–1071. 10.1016/j.bbagen.2013.10.035 24184936 PMC3909678

[jnc15198-bib-0093] Kamola, P. J. , Kitson, J. D. A. , Turner, G. , Maratou, K. , Eriksson, S. , Panjwani, A. , Warnock, L. C. , Douillard Guilloux, G. A. , Moores, K. , Koppe, E. L. , Wixted, W. E. , Wilson, P. A. , Gooderham, N. J. , Gant, T. W. , Clark, K. L. , Hughes, S. A. , Edbrooke, M. R. , & Parry, J. D. (2015). In silico and in vitro evaluation of exonic and intronic off‐target effects form a critical element of therapeutic ASO gapmer optimization. Nucleic Acids Research, 43, 8638–8650. 10.1093/nar/gkv857 26338776 PMC4605310

[jnc15198-bib-0094] Kanduri, C. (2008). Functional insights into long antisense noncoding RNA Kcnq1ot1 mediated bidirectional silencing. RNA Biology, 5, 208–211. 10.4161/rna.7113 18971626

[jnc15198-bib-0095] Kanning, K. C. , Kaplan, A. , & Henderson, C. E. (2010). Motor neuron diversity in development and disease. Annual Review of Neuroscience, 33, 409–440. 10.1146/annurev.neuro.051508.135722 20367447

[jnc15198-bib-0096] Karaki, S. , Paris, C. , & Rocchi, P. (2019). Antisense Oligonucleotides, A Novel Developing Targeting Therapy, in: Antisense Therapy. IntechOpen. 10.5772/intechopen.82105

[jnc15198-bib-0097] Keightley, M.‐C. , Carradice, D. P. , Layton, J. E. , Pase, L. , Bertrand, J. Y. , Wittig, J. G. , Dakic, A. , Badrock, A. P. , Cole, N. J. , Traver, D. , Nutt, S. L. , McCoey, J. , Buckle, A. M. , Heath, J. K. , & Lieschke, G. J. (2017). The Pu.1 target gene Zbtb11 regulates neutrophil development through its integrase‐like HHCC zinc finger. Nature Communications, 8, 14911. 10.1038/ncomms14911 PMC5384227 28382966

[jnc15198-bib-0098] Khorkova, O. , Myers, A. J. , Hsiao, J. , & Wahlestedt, C. (2014). Natural antisense transcripts. Human Molecular Genetics, 23, R54–R63. 10.1093/hmg/ddu207 24838284 PMC4170719

[jnc15198-bib-0099] Kovanda, A. , Zalar, M. , Šket, P. , Plavec, J. , & Rogelj, B. (2015). Anti‐sense DNA d(GGCCCC)n expansions in C9ORF72 form i‐motifs and protonated hairpins. Scientific Reports, 5, 3–9. 10.1038/srep17944 PMC4668579 26632347

[jnc15198-bib-0100] Kramer, N. J. , Carlomagno, Y. , Zhang, Y. J. , Almeida, S. , Cook, C. N. , Gendron, T. F. , Prudencio, M. , Van Blitterswijk, M. , Belzil, V. , Couthouis, J. , Paul, J. W. , Goodman, L. D. , Daughrity, L. , Chew, J. , Garrett, A. , Pregent, L. , Jansen‐West, K. , Tabassian, L. J. , Rademakers, R. , … Gitler, A. D. (2016). Spt4 selectively regulates the expression of C9orf72 sense and antisense mutant transcripts. Science, 353(6300), 708–712. 10.1126/science.aaf7791 27516603 PMC5823025

[jnc15198-bib-0101] Kung, J. T. Y. , Colognori, D. , & Lee, J. T. (2013). Long noncoding RNAs: Past, present, and future. Genetics, 193(3), 651–669. 10.1534/genetics.112.146704. 23463798 PMC3583990

[jnc15198-bib-0102] Kwon, I. , Xiang, S. , Kato, M. , Wu, L. , Theodoropoulos, P. , Wang, T. , Kim, J. , Yun, J. , Xie, Y. , & McKnight, S. L. (2014). Poly‐dipeptides encoded by the C9orf72 repeats bind nucleoli, impede RNA biogenesis, and kill cells. Science, 345, 1139–1145. 10.1126/science.1254917 25081482 PMC4459787

[jnc15198-bib-0103] Lagier‐Tourenne, C. , Baughn, M. , Rigo, F. , Sun, S. , Liu, P. , Li, H. R. , Jiang, J. , Watt, A. T. , Chun, S. , Katz, M. , Qiu, J. , Sun, Y. , Ling, S. C. , Zhu, Q. , Polymenidou, M. , Drenner, K. , Artates, J. W. , McAlonis‐Downes, M. , Markmiller, S. , … Ravits, J. (2013). Targeted degradation of sense and antisense C9orf72 RNA foci as therapy for ALS and frontotemporal degeneration. Proceedings of the National Academy of Sciences, 110, 10.1073/pnas.1318835110 PMC3839752 24170860

[jnc15198-bib-0104] Lee, J. K. , Shin, J. H. , Lee, J. E. , & Choi, E. J. (2015). Role of autophagy in the pathogenesis of amyotrophic lateral sclerosis. Biochimica Et Biophysica Acta (BBA) ‐ Molecular Basis of Disease, 1852(11), 2517–2524. 10.1016/j.bbadis.2015.08.005 26264610

[jnc15198-bib-0105] Lee, Y. B. , Chen, H. J. , Peres, J. N. , Gomez‐Deza, J. , Attig, J. , Štalekar, M. , Troakes, C. , Nishimura, A. L. , Scotter, E. L. , Vance, C. , Adachi, Y. , Sardone, V. , Miller, J. W. , Smith, B. N. , Gallo, J. M. , Ule, J. , Hirth, F. , Rogelj, B. , Houart, C. , & Shaw, C. E. (2013). Hexanucleotide repeats in ALS/FTD form length‐dependent RNA Foci, sequester RNA binding proteins, and are neurotoxic. Cell Reports, 5, 1178–1186. 10.1016/j.celrep.2013.10.049 24290757 PMC3898469

[jnc15198-bib-0106] Lefebvre, S. , Bürglen, L. , Reboullet, S. , Clermont, O. , Burlet, P. , Viollet, L. , Benichou, B. , Cruaud, C. , Millasseau, P. , Zeviani, M. , Le Paslier, D. , Frézal, J. , Cohen, D. , Weissenbach, J. , Munnich, A. , & Melki, J. (1995). Identification and characterization of a spinal muscular atrophy‐determining gene. Cell, 80, 155–165. 10.1016/0092-8674(95)90460-3 7813012

[jnc15198-bib-0107] Lelli, A. , Nolan, K. , Santambrogio, S. , Marti, H. , Hoogewijs, D. , Frew, I. , Goncalves, A. F. , Schonenberger, M. , Guinot, A. , & Wenger, R. (2015). Induction of long noncoding RNA MALAT1 in hypoxic mice. Hypoxia, 3, 45, 10.2147/HP.S90555 27774481 PMC5045088

[jnc15198-bib-0108] Li, P. P. , Sun, X. , Xia, G. , Arbez, N. , Paul, S. , Zhu, S. , Peng, H. B. , Ross, C. A. , Koeppen, A. H. , Margolis, R. L. , Pulst, S. M. , Ashizawa, T. , & Rudnicki, D. D. (2016). ATXN2‐AS, a gene antisense to ATXN2, is associated with spinocerebellar ataxia type 2 and amyotrophic lateral sclerosis. Annals of Neurology, 80, 600–615. 10.1002/ana.24761 27531668 PMC6555153

[jnc15198-bib-0109] Li, T. , Xie, J. , Shen, C. , Cheng, D. , Shi, Y. , Wu, Z. , Deng, X. , Chen, H. , Shen, B. , Peng, C. , Li, H. , Zhan, Q. , & Zhu, Z. (2016). Upregulation of long noncoding RNA ZEB1‐AS1 promotes tumor metastasis and predicts poor prognosis in hepatocellular carcinoma. Oncogene, 35, 1575–1584. 10.1038/onc.2015.223 26073087

[jnc15198-bib-0110] Li, X. , Yang, L. , & Chen, L. L. (2018). The biogenesis, functions, and challenges of circular RNAs. Molecular Cell, 71(3), 428–442. 10.1016/j.molcel.2018.06.034 30057200

[jnc15198-bib-0111] Lipovich, L. , Dachet, F. , Cai, J. , Bagla, S. , Balan, K. , Jia, H. , & Loeb, J. A. (2012). Activity‐dependent human brain coding/noncoding gene regulatory networks. Genetics, 192, 1133–1148. 10.1534/genetics.112.145128 22960213 PMC3522156

[jnc15198-bib-0112] Liu, Y. , Pattamatta, A. , Zu, T. , Reid, T. , Bardhi, O. , Borchelt, D. R. , Yachnis, A. T. , & Ranum, L. P. W. (2016). C9orf72 BAC mouse model with motor deficits and neurodegenerative features of ALS/FTD. Neuron, 90, 521–534. 10.1016/j.neuron.2016.04.005 27112499

[jnc15198-bib-0113] Lo Piccolo, L. , & Yamaguchi, M. (2017). RNAi of arcRNA hsrω affects sub‐cellular localization of Drosophila FUS to drive neurodiseases. Experimental Neurology, 292, 125–134. 10.1016/j.expneurol.2017.03.011 28342748

[jnc15198-bib-0114] Lorson, C. L. , Hahnen, E. , Androphy, E. J. , & Wirth, B. (1999). A single nucleotide in the SMN gene regulates splicing and is responsible for spinal muscular atrophy. Proceedings of the National Academy of Sciences, 96, 6307–6311. 10.1073/pnas.96.11.6307 PMC26877 10339583

[jnc15198-bib-0115] Lu, Q. R. , Sun, T. , Zhu, Z. , Ma, N. , Garcia, M. , Stiles, C. D. , & Rowitch, D. H. (2002). Common developmental requirement for Olig function indicates a motor neuron/oligodendrocyte connection. Cell, 109, 75–86. 10.1016/S0092-8674(02)00678-5 11955448

[jnc15198-bib-0116] Lunn, M. , & Wang, C. (2016). Spinal muscular atrophy. Neurology, 86, 884–885. 10.1212/WNL.0000000000002453 26865521

[jnc15198-bib-0117] Ma, L. , Bajic, V. B. , & Zhang, Z. (2013). On the classification of long non‐coding RNAs. RNA Biology, 10, 924–933. 10.4161/rna.24604 PMC4111732 23696037

[jnc15198-bib-0118] Magaña, J. J. , Velázquez‐Pérez, L. , & Cisneros, B. (2013). Spinocerebellar ataxia Type 2: Clinical presentation, molecular mechanisms, and therapeutic perspectives. Molecular Neurobiology, 47(1), 90–104. 10.1007/s12035-012-8348-8 22996397

[jnc15198-bib-0119] Majounie, E. , Renton, A. E. , Mok, K. , Dopper, E. G. P. , Waite, A. , Rollinson, S. , Chiò, A. , Restagno, G. , Nicolaou, N. , Simon‐Sanchez, J. , van Swieten, J. C. , Abramzon, Y. , Johnson, J. O. , Sendtner, M. , Pamphlett, R. , Orrell, R. W. , Mead, S. , Sidle, K. C. , Houlden, H. , … Logroscino, G. (2012). Frequency of the C9orf72 hexanucleotide repeat expansion in patients with amyotrophic lateral sclerosis and frontotemporal dementia: A cross‐sectional study. The Lancet Neurology, 11, 323–330. 10.1016/S1474-4422(12)70043-1 22406228 PMC3322422

[jnc15198-bib-0120] Malecová, B. , & Morris, K. V. (2010). Transcriptional gene silencing through epigenetic changes mediated by non‐coding RNAs. Current Opinion in Molecular Therapeutics, 12(2), 214. 20373265 PMC2861437

[jnc15198-bib-0121] Managadze, D. , Lobkovsky, A. E. , Wolf, Y. I. , Shabalina, S. A. , Rogozin, I. B. , & Koonin, E. V. (2013). The vast, conserved mammalian lincRNome. PLoS Computational Biology, 9, e1002917. 10.1371/journal.pcbi.1002917 23468607 PMC3585383

[jnc15198-bib-0122] Mao, Y. S. , Sunwoo, H. , Zhang, B. , & Spector, D. L. (2011). Direct visualization of the co‐transcriptional assembly of a nuclear body by noncoding RNAs. Nature Cell Biology, 13, 95–101. 10.1038/ncb2140 21170033 PMC3007124

[jnc15198-bib-0123] Marchese, F. P. , Raimondi, I. , & Huarte, M. (2017). The multidimensional mechanisms of long noncoding RNA function. Genome Biology, 18, 1–13. 10.1186/s13059-017-1348-2 29084573 PMC5663108

[jnc15198-bib-0124] Mehler, M. F. , & Mattick, J. S. (2007). Noncoding RNAs and RNA editing in brain development, functional diversification, and neurological disease. Physiological Reviews, 87, 799–823. 10.1152/physrev.00036.2006 17615389

[jnc15198-bib-0125] Memczak, S. , Jens, M. , Elefsinioti, A. , & Torti, F. (2013). Circular RNAs are a large class of animal RNAs with regulatory potency. Nature, 495, 333–338. 10.1038/nature11928 23446348

[jnc15198-bib-0126] Meng, S. , Zhou, H. , Feng, Z. , Xu, Z. , Tang, Y. , & Wu, M. (2019). Epigenetics in neurodevelopment: Emerging role of circular RNA. Frontiers in Cellular Neuroscience, 13, 1–12. 10.3389/fncel.2019.00327 31379511 PMC6658887

[jnc15198-bib-0127] Mercer, T. R. , Dinger, M. E. , Sunkin, S. M. , Mehler, M. F. , & Mattick, J. S. (2008). Specific expression of long noncoding RNAs in the mouse brain. Proceedings of the National Academy of Sciences, 105(2), 716–721. 10.1073/pnas.0706729105 PMC2206602 18184812

[jnc15198-bib-0128] Michalik, K. M. , You, X. , Manavski, Y. , Doddaballapur, A. , Zörnig, M. , Braun, T. , John, D. , Ponomareva, Y. , Chen, W. , Uchida, S. , Boon, R. A. , & Dimmeler, S. (2014). Long noncoding RNA MALAT1 regulates endothelial cell function and vessel growth. Circulation Research, 114, 1389–1397. 10.1161/CIRCRESAHA.114.303265 24602777

[jnc15198-bib-0129] Michelhaugh, S. K. , Lipovich, L. , Blythe, J. , Jia, H. , Kapatos, G. , & Bannon, M. J. (2011). Mining Affymetrix microarray data for long non‐coding RNAs: Altered expression in the nucleus accumbens of heroin abusers. Journal of Neurochemistry, 116, 459–466. 10.1111/j.1471-4159.2010.07126.x 21128942 PMC3061462

[jnc15198-bib-0130] Militello, G. , Hosen, M. R. , Ponomareva, Y. , Gellert, P. , Weirick, T. , John, D. , Hindi, S. M. , Mamchaoui, K. , Mouly, V. , Döring, C. , Zhang, L. , Nakamura, M. , Kumar, A. , Fukada, S. I. , Dimmeler, S. , & Uchida, S. (2018). A novel long non‐coding RNA Myolinc regulates myogenesis through TDP‐43 and Filip1. Journal of Molecular Cell Biology, 10, 102–117. 10.1093/jmcb/mjy025 29618024 PMC7191624

[jnc15198-bib-0131] Mizielinska, S. , Grönke, S. , Niccoli, T. , Ridler, C. E. , Clayton, E. L. , Devoy, A. , Moens, T. , Norona, F. E. , Woollacott, I. O. C. , Pietrzyk, J. , Cleverley, K. , Nicoll, A. J. , Pickering‐Brown, S. , Dols, J. , Cabecinha, M. , Hendrich, O. , Fratta, P. , Fisher, E. M. C. , Partridge, L. , & Isaacs, A. M. (2014). C9orf72 repeat expansions cause neurodegeneration in Drosophila through arginine‐rich proteins. Science, 345, 1192–1194. 10.1126/science.1256800 25103406 PMC4944841

[jnc15198-bib-0132] Mizielinska, S. , Lashley, T. , Norona, F. E. , Clayton, E. L. , Ridler, C. E. , Fratta, P. , & Isaacs, A. M. (2013). C9orf72 frontotemporal lobar degeneration is characterised by frequent neuronal sense and antisense RNA foci. Acta Neuropathologica, 126, 845–857. 10.1007/s00401-013-1200-z 24170096 PMC3830745

[jnc15198-bib-0133] Modarresi, F. , Faghihi, M. A. , Lopez‐Toledano, M. A. , Fatemi, R. P. , Magistri, M. , Brothers, S. P. , Van Der Brug, M. P. , & Wahlestedt, C. (2012). Inhibition of natural antisense transcripts in vivo results in gene‐specific transcriptional upregulation. Nature Biotechnology, 30, 453–459. 10.1038/nbt.2158 PMC4144683 22446693

[jnc15198-bib-0134] Moens, T. G. , Mizielinska, S. , Niccoli, T. , Mitchell, J. S. , Thoeng, A. , Ridler, C. E. , Grönke, S. , Esser, J. , Heslegrave, A. , Zetterberg, H. , Partridge, L. , & Isaacs, A. M. (2018). Sense and antisense RNA are not toxic in Drosophila models of C9orf72‐associated ALS/FTD. Acta Neuropathologica, 135, 445–457. 10.1007/s00401-017-1798-3 29380049 PMC6385858

[jnc15198-bib-0135] Moens, T. G. , Partridge, L. , & Isaacs, A. M. (2017). Genetic models of C9orf72: What is toxic? Current Opinion in Genetics and Development, 44, 92–101. 10.1016/j.gde.2017.01.006 28364657

[jnc15198-bib-0136] Monani, U. R. , Lorson, C. L. , Parsons, D. W. , Prior, T. W. , Androphy, E. J. , Burghes, A. H. M. , & McPherson, J. D. (1999). A single nucleotide difference that alters splicing patterns distinguishes the SMA gene SMN1 from the copy gene SMN2. Human Molecular Genetics, 8, 1177–1183. 10.1093/hmg/8.7.1177 10369862

[jnc15198-bib-0137] Monia, B. P. , Johnston, J. F. , Ecker, D. J. , Zounes, M. A. , Lima, W. F. , & Freier, S. M. (1992). Selective inhibition of mutant Ha‐ras mRNA expression by antisense oligonucleotides. Journal of Biological Chemistry, 267, 19954–19962. 1400312

[jnc15198-bib-0138] Mori, K. , Arzberger, T. , Grässer, F. A. , Gijselinck, I. , May, S. , Rentzsch, K. , Weng, S. M. , Schludi, M. H. , Van Der Zee, J. , Cruts, M. , Van Broeckhoven, C. , Kremmer, E. , Kretzschmar, H. A. , Haass, C. , & Edbauer, D. (2013). Bidirectional transcripts of the expanded C9orf72 hexanucleotide repeat are translated into aggregating dipeptide repeat proteins. Acta Neuropathologica, 126, 881–893. 10.1007/s00401-013-1189-3 24132570

[jnc15198-bib-0139] Mori, K. , Weng, S. M. , Arzberger, T. , May, S. , Rentzsch, K. , Kremmer, E. , Schmid, B. , Kretzschmar, H. A. , Cruts, M. , Van Broeckhoven, C. , Haass, C. , & Edbauer, D. (2013). The C9orf72 GGGGCC repeat is translated into aggregating dipeptide‐repeat proteins in FTLD/ALS. Science, 339, 1335–1338. 10.1126/science.1232927 23393093

[jnc15198-bib-0140] Naganuma, T. , Nakagawa, S. , Tanigawa, A. , Sasaki, Y. F. , Goshima, N. , & Hirose, T. (2012). Alternative 3′‐end processing of long noncoding RNA initiates construction of nuclear paraspeckles. EMBO Journal, 31, 4020–4034. 10.1038/emboj.2012.251 PMC3474925 22960638

[jnc15198-bib-0141] Nishimoto, Y. , Nakagawa, S. , Hirose, T. , Okano, H. J. , Takao, M. , Shibata, S. , Suyama, S. , Kuwako, K. I. , Imai, T. , Murayama, S. , Suzuki, N. , & Okano, H. (2013). The long non‐coding RNA nuclear‐enriched abundant transcript 1–2 induces paraspeckle formation in the motor neuron during the early phase of amyotrophic lateral sclerosis. Molecular Brain, 6, 1–18. 10.1186/1756-6606-6-31 23835137 PMC3729541

[jnc15198-bib-0142] Nishimura, A. L. , Mitne‐Neto, M. , Silva, H. C. A. , Richieri‐Costa, A. , Middleton, S. , Cascio, D. , Kok, F. , Oliveira, J. R. M. , Gillingwater, T. , Webb, J. , Skehel, P. , & Zatz, M. (2004). A mutation in the vesicle‐trafficking protein VAPB causes late‐onset spinal muscular atrophy and amyotrophic lateral sclerosis. American Journal of Human Genetics, 75, 822–831. 10.1086/425287 15372378 PMC1182111

[jnc15198-bib-0143] Noh, J. H. , Kim, K. M. , McClusky, W. G. , Abdelmohsen, K. , & Gorospe, M. (2018). Cytoplasmic functions of long noncoding RNAs. Wiley Interdisciplinary Reviews: RNA, 9, e1471. 10.1002/wrna.1471 29516680 PMC5963534

[jnc15198-bib-0144] O’Rourke, J. G. , Bogdanik, L. , Muhammad, A. K. M. G. , Gendron, T. F. , Kim, K. J. , Austin, A. , Cady, J. , Liu, E. Y. , Zarrow, J. , Grant, S. , Ho, R. , Bell, S. , Carmona, S. , Simpkinson, M. , Lall, D. , Wu, K. , Daughrity, L. , Dickson, D. W. , Harms, M. B. , … Baloh, R. H. (2015). C9orf72 BAC transgenic mice display typical pathologic features of ALS/FTD. Neuron, 88, 892–901. 10.1016/j.neuron.2015.10.027 26637796 PMC4672384

[jnc15198-bib-0145] Ogata, T. , & Kagami, M. (2016). Kagami‐Ogata syndrome: A clinically recognizable upd(14)pat and related disorder affecting the chromosome 14q32.2 imprinted region. Journal of Human Genetics, 61(2), 87–94. 10.1038/jhg.2015.113 26377239 PMC4771937

[jnc15198-bib-0146] Ottesen, E. W. , Luo, D. , Seo, J. , Singh, N. N. , & Singh, R. N. (2019). Human survival motor neuron genes generate a vast repertoire of circular RNAs. Nucleic Acids Research, 47, 2884–2905. 10.1093/nar/gkz034 30698797 PMC6451121

[jnc15198-bib-0147] Palazzo, A. F. , & Lee, E. S. (2015). Non‐coding RNA: What is functional and what is junk? Front. Genet., 5, 2. 10.3389/fgene.2015.00002 PMC4306305 25674102

[jnc15198-bib-0148] Papaioannou, D. , Petri, A. , Dovey, O. M. , Terreri, S. , Wang, E. , Collins, F. A. , Woodward, L. A. , Walker, A. E. , Nicolet, D. , Pepe, F. , Kumchala, P. , Bill, M. , Walker, C. J. , Karunasiri, M. , Mrózek, K. , Gardner, M. L. , Camilotto, V. , Zitzer, N. , Cooper, J. L. , … Garzon, R. (2019). The long non‐coding RNA HOXB‐AS3 regulates ribosomal RNA transcription in NPM1‐mutated acute myeloid leukemia. Nature Communications, 10, 1–15. 10.1038/s41467-019-13259-2 PMC6877618 31767858

[jnc15198-bib-0149] Park, H. C. , Mehta, A. , Richardson, J. S. , & Appel, B. (2002). olig2 is required for zebrafish primary motor neuron and oligodendrocyte development. Developmental Biology, 248, 356–368. 10.1006/dbio.2002.0738 12167410

[jnc15198-bib-0150] Pereira Fernandes, D. , Bitar, M. , Jacobs, F. , & Barry, G. (2018). Long non‐coding RNAs in neuronal aging. Non‐Coding RNA, 4, 12. 10.3390/ncrna4020012 PMC6027360 29670042

[jnc15198-bib-0151] Peters, O. M. , Cabrera, G. T. , Tran, H. , Gendron, T. F. , McKeon, J. E. , Metterville, J. , Weiss, A. , Wightman, N. , Salameh, J. , Kim, J. , Sun, H. , Boylan, K. B. , Dickson, D. , Kennedy, Z. , Lin, Z. , Zhang, Y. J. , Daughrity, L. , Jung, C. , Gao, F. B. , … Brown, R. H. (2015). Human C9ORF72 hexanucleotide expansion reproduces RNA foci and dipeptide repeat proteins but not neurodegeneration in BAC transgenic mice. Neuron, 88, 902–909. 10.1016/j.neuron.2015.11.018 26637797 PMC4828340

[jnc15198-bib-0152] Polikepahad, S. , & Corry, D. B. (2013). Profiling of T helper cell‐derived small RNAs reveals unique antisense transcripts and differential association of miRNAs with argonaute proteins 1 and 2. Nucleic Acids Research, 41, 1164–1177. 10.1093/nar/gks1098 23185045 PMC3553939

[jnc15198-bib-0153] Ponjavic, J. , Oliver, P. L. , Lunter, G. , & Ponting, C. P. (2009). Genomic and transcriptional co‐localization of protein‐coding and long non‐coding RNA pairs in the developing brain. PLoS Genetics, 5, e1000617. 10.1371/journal.pgen.1000617 19696892 PMC2722021

[jnc15198-bib-0154] Quan, Z. , Zheng, D. , & Qing, H. (2017). Regulatory roles of long non‐coding RNAs in the central nervous system and associated neurodegenerative diseases. Frontiers in Cellular Neuroscience, 11, 175. 10.3389/fncel.2017.00175 28713244 PMC5491930

[jnc15198-bib-0155] Quinn, J. J. , & Chang, H. Y. (2016). Unique features of long non‐coding RNA biogenesis and function. Nature Reviews Genetics, 17, 47–62. 10.1038/nrg.2015.10 26666209

[jnc15198-bib-0156] Qureshi, I. A. , & Mehler, M. F. (2012). Emerging roles of non‐coding RNAs in brain evolution, development, plasticity and disease. Nature Reviews Neuroscience, 13, 528–541. 10.1038/nrn3234 22814587 PMC3478095

[jnc15198-bib-0157] Rademakers, R. (2012). C9orf72 repeat expansions in patients with ALS and FTD. The Lancet Neurology, 11, 297–298. 10.1016/S1474-4422(12)70046-7 22406229 PMC4114244

[jnc15198-bib-0158] Ransohoff, J. D. , Wei, Y. , & Khavari, P. A. (2018). functions and unique features of long intergenic non‐coding RNA. Nature Reviews Molecular Cell Biology, 19(3), 143–157. 10.1038/nrm.2017.104 29138516 PMC5889127

[jnc15198-bib-0159] Rashid, F. , Shah, A. , & Shan, G. (2016). Long non‐coding RNAs in the cytoplasm. Genomics, Proteomics and Bioinformatics, 14, 73–80. 10.1016/j.gpb.2016.03.005 PMC4880952 27163185

[jnc15198-bib-0160] Ravanelli, A. M. , & Appel, B. (2015). Motor neurons and oligodendrocytes arise from distinct cell lineages by progenitor recruitment. Genes and Development, 29, 2504–2515. 10.1101/gad.271312.115 26584621 PMC4691953

[jnc15198-bib-0161] Ravasi, T. , Suzuki, H. , Pang, K. C. , Katayama, S. , Furuno, M. , Okunishi, R. , Fukuda, S. , Ru, K. , Frith, M. C. , Gongora, M. M. , Grimmond, S. M. , Hume, D. A. , Hayashizaki, Y. , & Mattick, J. S. (2006). Experimental validation of the regulated expression of large numbers of non‐coding RNAs from the mouse genome. Genome Research, 16, 11–19. 10.1101/gr.4200206 16344565 PMC1356124

[jnc15198-bib-0162] Ray, M. K. , Wiskow, O. , King, M. J. , Ismail, N. , Ergun, A. , Wang, Y. , Plys, A. J. , Davis, C. P. , Kathrein, K. , Sadreyev, R. , Borowsky, M. L. , Eggan, K. , Zon, L. , Galloway, J. L. , & Kingston, R. E. (2016). CAT7 and cat7l long non‐coding RNAs tune polycomb repressive complex 1 function during human and zebrafish development. Journal of Biological Chemistry, 291, 19558–19572. 10.1074/jbc.M116.730853 PMC5016691 27405765

[jnc15198-bib-0163] Reddy, K. , Zamiri, B. , Stanley, S. Y. R. , Macgregor, R. B. , & Pearson, C. E. (2013). The disease‐associated r(GGGGCC)n repeat from the C9orf72 gene forms tract length‐dependent uni‐ and multimolecular RNA G‐quadruplex structures. Journal of Biological Chemistry, 288, 9860–9866. 10.1074/jbc.C113.452532 PMC3617286 23423380

[jnc15198-bib-0164] Renton, A. E. , Chiò, A. , & Traynor, B. J. (2014). State of play in amyotrophic lateral sclerosis genetics. Nature Neuroscience, 17, 17–23. 10.1038/nn.3584 24369373 PMC4544832

[jnc15198-bib-0165] Renton, A. E. , Majounie, E. , Waite, A. , Simón‐Sánchez, J. , Rollinson, S. , Gibbs, J. R. , Schymick, J. C. , Laaksovirta, H. , van Swieten, J. C. , Myllykangas, L. , Kalimo, H. , Paetau, A. , Abramzon, Y. , Remes, A. M. , Kaganovich, A. , Scholz, S. W. , Duckworth, J. , Ding, J. , Harmer, D. W. , … Traynor, B. J. (2011). A hexanucleotide repeat expansion in C9ORF72 is the cause of chromosome 9p21‐linked ALS‐FTD. Neuron, 72, 257–268. 10.1016/j.neuron.2011.09.010 21944779 PMC3200438

[jnc15198-bib-0166] Rinn, J. L. , & Chang, H. Y. (2012). Genome regulation by long noncoding RNAs. Annual Review of Biochemistry, 81, 145–166. 10.1146/annurev-biochem-051410-092902 PMC3858397 22663078

[jnc15198-bib-0167] Rinn, J. L. , Kertesz, M. , Wang, J. K. , Squazzo, S. L. , Xu, X. , Brugmann, S. A. , Goodnough, L. H. , Helms, J. A. , Farnham, P. J. , Segal, E. , & Chang, H. Y. (2007). Functional demarcation of active and silent chromatin domains in human HOX loci by noncoding RNAs. Cell, 10.1016/j.cell.2007.05.022 PMC2084369 17604720

[jnc15198-bib-0168] Rizvi, A. , Camara, P. G. , Kandror, E. K. , Roberts, T. J. , Schieren, I. , Maniatis, T. , & Rabadan, R. (2017). Single‐cell topological RNA‐seq analysis reveals insights into cellular differentiation and development. Nature Biotechnology, 35, 551–560. 10.1038/nbt.3854 PMC5569300 28459448

[jnc15198-bib-0169] Robberecht, W. , & Philips, T. (2013). The changing scene of amyotrophic lateral sclerosis. Nature Reviews Neuroscience, 14, 248–264. 10.1038/nrn3430 23463272

[jnc15198-bib-0170] Rybak‐Wolf, A. , Stottmeister, C. , Glažar, P. , Jens, M. , Pino, N. , Hanan, M. , Behm, M. , Bartok, O. , Ashwal‐Fluss, R. , Herzog, M. , Schreyer, L. , Papavasileiou, P. , Ivanov, A. , Öhman, M. , Refojo, D. , Kadener, S. , & Rajewsky, N. (2015). Circular RNAs in the mammalian brain are highly abundant, conserved, and dynamically expressed. Molecular Cell, 58, 870–885. 10.1016/j.molcel.2015.03.027 25921068

[jnc15198-bib-0171] Saberi, S. , Stauffer, J. E. , Jiang, J. , Garcia, S. D. , Taylor, A. E. , Schulte, D. , Ohkubo, T. , Schloffman, C. L. , Maldonado, M. , Baughn, M. , Rodriguez, M. J. , Pizzo, D. , Cleveland, D. , & Ravits, J. (2018). Sense‐encoded poly‐GR dipeptide repeat proteins correlate to neurodegeneration and uniquely co‐localize with TDP‐43 in dendrites of repeat‐expanded C9orf72 amyotrophic lateral sclerosis. Acta Neuropathologica, 135, 459–474. 10.1007/s00401-017-1793-8 29196813 PMC5935138

[jnc15198-bib-0172] Salzman, J. (2016). Circular RNA expression: Its potential regulation and function. Trends in Genetics, 32, 309–316. 10.1016/j.tig.2016.03.002 27050930 PMC4948998

[jnc15198-bib-0173] Salzman, J. , Gawad, C. , Wang, P. L. , Lacayo, N. , & Brown, P. O. (2012). Circular RNAs are the predominant transcript isoform from hundreds of human genes in diverse cell types. PLoS One, 7, e30733. 10.1371/journal.pone.0030733 22319583 PMC3270023

[jnc15198-bib-0174] Sareen, D. , O’Rourke, J. G. , Meera, P. , Muhammad, A. K. M. G. , Grant, S. , Simpkinson, M. , Bell, S. , Carmona, S. , Ornelas, L. , Sahabian, A. , Gendron, T. , Petrucelli, L. , Baughn, M. , Ravits, J. , Harms, M. B. , Rigo, F. , Bennett, C. F. , Otis, T. S. , Svendsen, C. N. , & Baloh, R. H. (2013). Targeting RNA foci in iPSC‐derived motor neurons from ALS patients with a C9ORF72 repeat expansion. Science Translational Medicine, 5, 208ra149. 10.1126/scitranslmed.3007529 PMC4090945 24154603

[jnc15198-bib-0175] Satterfield, T. F. , & Pallanck, L. J. (2006). Ataxin‐2 and its Drosophila homolog, ATX2, physically assemble with polyribosomes. Human Molecular Genetics, 15, 2523–2532. 10.1093/hmg/ddl173 16835262

[jnc15198-bib-0176] Schoch, K. M. , & Miller, T. M. (2017). Antisense oligonucleotides: Translation from mouse models to human neurodegenerative diseases. Neuron, 94, 1056–1070. 10.1016/j.neuron.2017.04.010 28641106 PMC5821515

[jnc15198-bib-0177] Scoles, D. R. , Minikel, E. V. , & Pulst, S. M. (2019). Antisense oligonucleotides. Neurology Genetics, 5, e323. 10.1212/NXG.0000000000000323 31119194 PMC6501637

[jnc15198-bib-0178] Scoles, D. R. , & Pulst, S. M. (2018). Spinocerebellar ataxia type 2. Advances in experimental medicine and biology (pp. 175–195). Springer. 10.1007/978-3-319-71779-1_8 29427103

[jnc15198-bib-0179] Sekar, S. , & Liang, W. S. (2019). Circular RNA expression and function in the brain. Non‐coding RNA Research, 4, 23–29. 10.1016/j.ncrna.2019.01.001 30891534 PMC6404376

[jnc15198-bib-0180] Sewell, K. L. , Geary, R. S. , Baker, B. F. , Glover, J. M. , Mant, T. G. K. , Yu, R. Z. , Tami, J. A. , & Dorr, F. A. (2002). Phase I trial of ISIS 104838, a 2′‐methoxyethyl modified antisense oligonucleotide targeting tumor necrosis factor‐α. Journal of Pharmacology and Experimental Therapeutics, 303, 1334–1343. 10.1124/jpet.102.036749 12438559

[jnc15198-bib-0181] Shelkovnikova, T. A. , Kukharsky, M. S. , An, H. , Dimasi, P. , Alexeeva, S. , Shabir, O. , Heath, P. R. , & Buchman, V. L. (2018). Protective paraspeckle hyper‐assembly downstream of TDP‐43 loss of function in amyotrophic lateral sclerosis. Molecular Neurodegeneration, 13, 1–17. 10.1186/s13024-018-0263-7 29859124 PMC5984788

[jnc15198-bib-0182] Shelkovnikova, T. A. , Robinson, H. K. , Troakes, C. , Ninkina, N. , & Buchman, V. L. (2014). Compromised paraspeckle formation as a pathogenic factor in FUSopathies. Human Molecular Genetics, 23, 2298–2312. 10.1093/hmg/ddt622 24334610 PMC3976330

[jnc15198-bib-0183] Shi, C. , Zhang, L. , & Qin, C. (2017). Long non‐coding RNAs in brain development, synaptic biology, and Alzheimer’s disease. Brain Research Bulletin, 132, 160–169. 10.1016/j.brainresbull.2017.03.010 28347717

[jnc15198-bib-0184] Simchovitz, A. , Hanan, M. , Niederhoffer, N. , Madrer, N. , Yayon, N. , Bennett, E. R. , Greenberg, D. S. , Kadener, S. , & Soreq, H. (2019). NEAT1 is overexpressed in Parkinson’s disease substantia nigra and confers drug‐inducible neuroprotection from oxidative stress. The FASEB Journal, 33, 11223–11234. 10.1096/fj.201900830R 31311324 PMC6766647

[jnc15198-bib-0185] Simone, R. , Balendra, R. , Moens, T. G. , Preza, E. , Wilson, K. M. , Heslegrave, A. , Woodling, N. S. , Niccoli, T. , Gilbert‐Jaramillo, J. , Abdelkarim, S. , Clayton, E. L. , Clarke, M. , Konrad, M. , Nicoll, A. J. , Mitchell, J. S. , Calvo, A. , Chio, A. , Houlden, H. , Polke, J. M. , … Isaacs, A. M. (2018). G‐quadruplex‐binding small molecules ameliorate C9orf72 FTD/ALS pathology in vitro and in vivo. EMBO Molecular Medicine, 10, 22–31. 10.15252/emmm.201707850 29113975 PMC5760849

[jnc15198-bib-0186] Sone, M. , Hayashi, T. , Tarui, H. , Agata, K. , Takeichi, M. , & Nakagawa, S. (2007). The mRNA‐like noncoding RNA Gomafu constitutes a novel nuclear domain in a subset of neurons. Journal of Cell Science, 120, 2498–2506. 10.1242/jcs.009357 17623775

[jnc15198-bib-0187] Sun, Q. , Hao, Q. , & Prasanth, K. V. (2018). Nuclear long noncoding RNAs: Key regulators of gene expression. Trends in Genetics, 34(2), 142–157. 10.1016/j.tig.2017.11.005 29249332 PMC6002860

[jnc15198-bib-0188] Sunwoo, H. , Dinger, M. E. , Wilusz, J. E. , Amaral, P. P. , Mattick, J. S. , & Spector, D. L. (2009). Men ε/β nuclear‐retained non‐coding RNAs are up‐regulated upon muscle differentiation and are essential components of paraspeckles. Genome Research, 19, 347–359. 10.1101/gr.087775.108 19106332 PMC2661813

[jnc15198-bib-0189] Sunwoo, J. S. , Lee, S. T. , Im, W. , Lee, M. , Byun, J. I. , Jung, K. H. , Park, K. I. , Jung, K. Y. , Lee, S. K. , Chu, K. , & Kim, M. (2017). Altered expression of the long noncoding RNA NEAT1 in Huntington’s disease. Molecular Neurobiology, 54, 1577–1586. 10.1007/s12035-016-9928-9 27221610

[jnc15198-bib-0190] Suzuki, H. , Shibagaki, Y. , Hattori, S. , & Matsuoka, M. (2019). C9‐ALS/FTD‐linked proline–arginine dipeptide repeat protein associates with paraspeckle components and increases paraspeckle formation. Cell Death and Disease, 10, 746. 10.1038/s41419-019-1983-5 31582731 PMC6776546

[jnc15198-bib-0191] Swinnen, B. , Bento‐Abreu, A. , Gendron, T. F. , Boeynaems, S. , Bogaert, E. , Nuyts, R. , Timmers, M. , Scheveneels, W. , Hersmus, N. , Wang, J. , Mizielinska, S. , Isaacs, A. M. , Petrucelli, L. , Lemmens, R. , Van Damme, P. , Van Den Bosch, L. , & Robberecht, W. (2018). A zebrafish model for C9orf72 ALS reveals RNA toxicity as a pathogenic mechanism. Acta Neuropathologica, 135, 427–443. 10.1007/s00401-017-1796-5 29302778

[jnc15198-bib-0192] Swinnen, B. , Robberecht, W. , & Van Den Bosch, L. (2020). RNA toxicity in non‐coding repeat expansion disorders. EMBO Journal, 39, 1–23. 10.15252/embj.2018101112 PMC6939197 31721251

[jnc15198-bib-0193] Tao, Z. , Wang, H. , Xia, Q. , Li, K. E. , Li, K. , Jiang, X. , Xu, G. , Wang, G. , & Ying, Z. (2015). Nucleolar stress and impaired stress granule formation contribute to C9orf72 RAN translation‐induced cytotoxicity. Human Molecular Genetics, 24, 2426–2441. 10.1093/hmg/ddv005 25575510

[jnc15198-bib-0194] Tollervey, J. R. , Curk, T. , Rogelj, B. , Briese, M. , Cereda, M. , Kayikci, M. , König, J. , Hortobágyi, T. , Nishimura, A. L. , Župunski, V. , Patani, R. , Chandran, S. , Rot, G. , Zupan, B. , Shaw, C. E. , & Ule, J. (2011). Characterizing the RNA targets and position‐dependent splicing regulation by TDP‐43. Nature Neuroscience, 14, 452–458. 10.1038/nn.2778 21358640 PMC3108889

[jnc15198-bib-0195] Tung, Y. T. , Peng, K. C. , Chen, Y. C. , Yen, Y. P. , Chang, M. , Thams, S. , & Chen, J. A. (2019). Mir‐17∼92 Confers motor neuron subtype differential resistance to ALS‐associated degeneration. Cell Stem Cell, 25, 193–209.e7. 10.1016/j.stem.2019.04.016 31155482

[jnc15198-bib-0196] Ulitsky, I. , & Bartel, D. P. (2013). XLincRNAs: Genomics, evolution, and mechanisms. Cell, 10.1016/j.cell.2013.06.020 PMC3924787 23827673

[jnc15198-bib-0197] Ulitsky, I. , Shkumatava, A. , Jan, C. H. , Sive, H. , & Bartel, D. P. (2011). Conserved function of lincRNAs in vertebrate embryonic development despite rapid sequence evolution. Cell, 147, 1537–1550. 10.1016/j.cell.2011.11.055 22196729 PMC3376356

[jnc15198-bib-0198] Van Arensbergen, J. , García‐Hurtado, J. , Moran, I. , Maestro, M. A. , Xu, X. , Van De Casteele, M. , Skoudy, A. L. , Palassini, M. , Heimberg, H. , & Ferrer, J. (2010). Derepression of polycomb targets during pancreatic organogenesis allows insulin‐producing beta‐cells to adopt a neural gene activity program. Genome Research, 20, 722–732. 10.1101/gr.101709.109 20395405 PMC2877569

[jnc15198-bib-0199] Van den Heuvel, D. M. A. , Harschnitz, O. , van den Berg, L. H. , & Pasterkamp, R. J. (2014). Taking a risk: A therapeutic focus on ataxin‐2 in amyotrophic lateral sclerosis? Trends Mol. Med., 20, 25–35. 10.1016/j.molmed.2013.09.001 24140266

[jnc15198-bib-0200] van Es, M. A. , Hardiman, O. , Chio, A. , Al‐Chalabi, A. , Pasterkamp, R. J. , Veldink, J. H. , & van den Berg, L. H. (2017). Amyotrophic lateral sclerosis. Lancet, 390, 2084–2098. 10.1016/S0140-6736(17)31287-4 28552366

[jnc15198-bib-0201] van Rossum, D. , Verheijen, B. M. , & Pasterkamp, R. J. (2016). Circular RNAs: Novel regulators of neuronal development. Frontiers in Molecular Neuroscience, 9, 1–7. 10.3389/fnmol.2016.00074 27616979 PMC4999478

[jnc15198-bib-0202] Venø, M. T. , Hansen, T. B. , Venø, S. T. , Clausen, B. H. , Grebing, M. , Finsen, B. , Holm, I. E. , & Kjems, J. (2015). Spatio‐temporal regulation of circular RNA expression during porcine embryonic brain development. Genome Biology, 16, 245. 10.1186/s13059-015-0801-3 26541409 PMC4635978

[jnc15198-bib-0203] Verheijen, B. M. , & Pasterkamp, R. J. (2017). Commentary: FUS affects circular RNA expression in murine embryonic stem cell‐derived motor neurons. Frontiers in Molecular Neuroscience, 10, 412. 10.3389/fnmol.2017.00412 29311805 PMC5732946

[jnc15198-bib-0204] Villar‐Menéndez, I. , Porta, S. , Buira, S. P. , Pereira‐Veiga, T. , Díaz‐Sánchez, S. , Albasanz, J. L. , Ferrer, I. , Martín, M. , & Barrachina, M. (2014). Increased striatal adenosine A2A receptor levels is an early event in Parkinson’s disease‐related pathology and it is potentially regulated by miR‐34b. Neurobiology of Diseases, 69, 206–214. 10.1016/j.nbd.2014.05.030 24892887

[jnc15198-bib-0205] Vollmer, J. , Jepsen, J. S. , Uhlmann, E. , Schetter, C. , Jurk, M. , Wader, T. , Wüllner, M. , & Krieg, A. M. (2004). Modulation of CpG oligodeoxynucleotide‐mediated immune stimulation by locked nucleic acid (LNA). Oligonucleotides, 14, 23–31. 10.1089/154545704322988021 15104893

[jnc15198-bib-0206] Wang, F. , Liang, R. , Soibam, B. , Yang, J. , & Liu, Y. (2019). Coregulatory long non‐coding RNA and protein‐coding genes in serum starved cells. Biochimica Et Biophysica Acta (BBA) ‐ Gene Regulatory Mechanisms, 1862, 84–95. 10.1016/j.bbagrm.2018.11.004 30503397

[jnc15198-bib-0207] Wang, K. C. , & Chang, H. Y. (2011). Molecular mechanisms of long noncoding RNAs. Molecular Cell, 43, 904–914. 10.1016/j.molcel.2011.08.018 21925379 PMC3199020

[jnc15198-bib-0208] Wang, K. C. , Yang, Y. W. , Liu, B. , Sanyal, A. , Corces‐Zimmerman, R. , Chen, Y. , Lajoie, B. R. , Protacio, A. , Flynn, R. A. , Gupta, R. A. , Wysocka, J. , Lei, M. , Dekker, J. , Helms, J. A. , & Chang, H. Y. (2011). A long noncoding RNA maintains active chromatin to coordinate homeotic gene expression. Nature, 472, 120–126. 10.1038/nature09819 21423168 PMC3670758

[jnc15198-bib-0209] Wang, S. M. , Liu, G. Q. , Xian, H. B. , Si, J. L. , Qi, S. X. , & Yu, Y. P. (2019). LncRNA NEAT1 alleviates sepsis‐induced myocardial injury by regulating the TLR2/NF‐κB signaling pathway. European Review for Medical and Pharmacological Sciences, 23, 4898–4907. 10.26355/eurrev_201906_18078 31210324

[jnc15198-bib-0210] Wang, X. , Goodrich, K. J. , Conlon, E. G. , Gao, J. , Erbse, A. H. , Manley, J. L. , & Cech, T. R. (2019). C9orf72 and triplet repeat disorder RNAs: G‐quadruplex formation, binding to PRC2 and implications for disease mechanisms. RNA, 25, 935–947. 10.1261/rna.071191.119 31048495 PMC6633194

[jnc15198-bib-0211] Wang, X.‐M. , Li, X.‐M. , Song, N. , Zhai, H. , Gao, X.‐M. , & Yang, Y.‐N. (2019). Long non‐coding RNAs H19, MALAT1 and MIAT as potential novel biomarkers for diagnosis of acute myocardial infarction. Biomedicine and Pharmacotherapy, 118, 109208. 10.1016/j.biopha.2019.109208 31302423

[jnc15198-bib-0212] Wang, Z. , Xu, P. , Chen, B. , Zhang, Z. , Zhang, C. , Zhan, Q. , Huang, S. , Xia, Z. A. , & Peng, W. (2018). Identifying circRNA‐associated‐ceRNA networks in the hippocampus of Aß1‐42‐induced Alzheimer’s disease‐like rats using microarray analysis. Aging (Albany., NY), 10, 775–788. 10.18632/aging.101427. 29706607 PMC5940119

[jnc15198-bib-0213] Wen, X. , Tan, W. , Westergard, T. , Krishnamurthy, K. , Markandaiah, S. S. , Shi, Y. , Lin, S. , Shneider, N. A. , Monaghan, J. , Pandey, U. B. , Pasinelli, P. , Ichida, J. K. , & Trotti, D. (2014). Antisense proline‐arginine RAN dipeptides linked to C9ORF72‐ALS/FTD form toxic nuclear aggregates that initiate invitro and invivo neuronal death. Neuron, 84, 1213–1225. 10.1016/j.neuron.2014.12.010 25521377 PMC4632245

[jnc15198-bib-0214] Werner, A. , Cockell, S. , Falconer, J. , Carlile, M. , Alnumeir, S. , & Robinson, J. (2014). Contribution of natural antisense transcription to an endogenous siRNA signature in human cells. BMC Genomics, 15, 19. 10.1186/1471-2164-15-19 24410956 PMC3898206

[jnc15198-bib-0215] Westholm, J. O. , Miura, P. , Olson, S. , Shenker, S. , Joseph, B. , Sanfilippo, P. , Celniker, S. E. , Graveley, B. R. , & Lai, E. C. (2014). Genome‐wide analysis of drosophila circular RNAs reveals their structural and sequence properties and age‐dependent neural accumulation. Cell Reports, 9, 1966–1980. 10.1016/j.celrep.2014.10.062 25544350 PMC4279448

[jnc15198-bib-0216] Wight, M. , & Werner, A. (2013). The functions of natural antisense transcripts. Essays in Biochemistry, 54, 91–101. 10.1042/BSE0540091 23829529 PMC4284957

[jnc15198-bib-0217] Wijesekera, L. C. , & Leigh, P. N. (2009). Amyotrophic lateral sclerosis. Orphanet Journal of Rare Diseases, 4, 3. 10.1186/1750-1172-4-3 19192301 PMC2656493

[jnc15198-bib-0218] Woo, C. J. , Maier, V. K. , Davey, R. , Brennan, J. , Li, G. , Brothers, J. , Schwartz, B. , Gordo, S. , Kasper, A. , Okamoto, T. R. , Johansson, H. E. , Mandefro, B. , Sareen, D. , Bialek, P. , Chau, B. N. , Bhat, B. , Bullough, D. , & Barsoum, J. (2017). Gene activation of SMN by selective disruption of lncRNA‐mediated recruitment of PRC2 for the treatment of spinal muscular atrophy. Proceedings of the National Academy of Sciences, 114, E1509–E1518. 10.1073/pnas.1616521114 PMC5338378 28193854

[jnc15198-bib-0219] Wu, L. , Liu, C. , & Zhang, Z. (2020). Knockdown of lncRNA MIAT inhibits proliferation and cisplatin resistance in non‐small cell lung cancer cells by increasing miR‐184 expression. Oncology Letters, 19, 533–541. 10.3892/ol.2019.11084 31897168 PMC6924112

[jnc15198-bib-0220] Xiao, S. , Sanelli, T. , Dib, S. , Sheps, D. , Findlater, J. , Bilbao, J. , Keith, J. , Zinman, L. , Rogaeva, E. , & Robertson, J. (2011). RNA targets of TDP‐43 identified by UV‐CLIP are deregulated in ALS. Molecular and Cellular Neurosciences, 47, 167–180. 10.1016/j.mcn.2011.02.013 21421050

[jnc15198-bib-0221] Xu, Z. , Poidevin, M. , Li, X. , Li, Y. , Shu, L. , Nelson, D. L. , Li, H. , Hales, C. M. , Gearing, M. , Wingo, T. S. , & Jin, P. (2013). Expanded GGGGCC repeat RNA associated with amyotrophic lateral sclerosis and frontotemporal dementia causes neurodegeneration. Proceedings of the National Academy of Sciences, 110, 7778–7783. 10.1073/pnas.1219643110 PMC3651485 23553836

[jnc15198-bib-0222] Yamazaki, T. , & Hirose, T. (2015). The building process of the functional paraspeckle with long non‐coding RNAs. Frontiers in Bioscience, 7, 1–47. 10.2741/715 25553361

[jnc15198-bib-0223] Yamazaki, T. , Souquere, S. , Chujo, T. , Kobelke, S. , Chong, Y. S. , Fox, A. H. , Bond, C. S. , Nakagawa, S. , Pierron, G. , & Hirose, T. (2018). Functional domains of NEAT1 architectural lncRNA induce paraspeckle assembly through phase separation. Molecular Cell, 70, 1038–1053.e7. 10.1016/j.molcel.2018.05.019 29932899

[jnc15198-bib-0224] Yan, L. , Yang, M. , Guo, H. , Yang, L. , Wu, J. , Li, R. , Liu, P. , Lian, Y. , Zheng, X. , Yan, J. , Huang, J. , Li, M. , Wu, X. , Wen, L. , Lao, K. , Li, R. , Qiao, J. , & Tang, F. (2013). Single‐cell RNA‐Seq profiling of human preimplantation embryos and embryonic stem cells. Nature Structural and Molecular Biology, 20, 1131–1139. 10.1038/nsmb.2660 23934149

[jnc15198-bib-0225] Yen, Y.‐P. , Hsieh, W.‐F. , Tsai, Y.‐Y. , Lu, Y.‐L. , Liau, E. S. , Hsu, H.‐C. , Chen, Y.‐C. , Liu, T.‐C. , Chang, M. , Li, J. , Lin, S.‐P. , Hung, J.‐H. , & Chen, J.‐A. (2018). Dlk1‐Dio3 locus‐derived lncRNAs perpetuate postmitotic motor neuron cell fate and subtype identity. Elife, 7, 1–35. 10.7554/eLife.38080 PMC6221546 30311912

[jnc15198-bib-0226] Yokoshi, M. , Li, Q. , Yamamoto, M. , Okada, H. , Suzuki, Y. , & Kawahara, Y. (2014). Direct binding of Ataxin‐2 to distinct elements in 3’ UTRs promotes mRNA stability and protein expression. Molecular Cell, 55, 186–198. 10.1016/j.molcel.2014.05.022 24954906

[jnc15198-bib-0227] You, X. , Vlatkovic, I. , Babic, A. , Will, T. , Epstein, I. , Tushev, G. , Akbalik, G. , Wang, M. , Glock, C. , Quedenau, C. , Wang, X. , Hou, J. , Liu, H. , Sun, W. , Sambandan, S. , Chen, T. , Schuman, E. M. , & Chen, W. (2015). Neural circular RNAs are derived from synaptic genes and regulated by development and plasticity. Nature Neuroscience, 18(4), 603–610. 10.1038/nn.3975 25714049 PMC4376664

[jnc15198-bib-0228] Yu, W. , Gius, D. , Onyango, P. , Muldoon‐Jacobs, K. , Karp, J. , Feinberg, A. P. , & Cui, H. (2008). Epigenetic silencing of tumour suppressor gene p15 by its antisense RNA. Nature, 451, 202–206. 10.1038/nature06468 18185590 PMC2743558

[jnc15198-bib-0229] Yuan, P. , Cao, W. , Zang, Q. , Li, G. , Guo, X. , & Fan, J. (2016). The HIF‐2α‐MALAT1‐miR‐216b axis regulates multi‐drug resistance of hepatocellular carcinoma cells via modulating autophagy. Biochemical and Biophysical Research Communications, 478, 1067–1073. 10.1016/j.bbrc.2016.08.065 27524242

[jnc15198-bib-0230] Yunusov, D. , Anderson, L. , DaSilva, L. F. , Wysocka, J. , Ezashi, T. , Roberts, R. M. , & Verjovski‐Almeida, S. (2016). HIPSTR and thousands of lncRNAs are heterogeneously expressed in human embryos, primordial germ cells and stable cell lines. Scientific Reports, 6, 32753. 10.1038/srep32753 27605307 PMC5015059

[jnc15198-bib-0231] Zamiri, B. , Reddy, K. , Macgregor, R. B. , & Pearson, C. E. (2014). TMPyP4 porphyrin distorts RNA G‐quadruplex structures of the disease‐associated r(GGGGCC)n repeat of the C9orf72 gene and blocks interaction of RNAbinding proteins. Journal of Biological Chemistry, 289, 4653–4659. 10.1074/jbc.C113.502336 PMC3931028 24371143

[jnc15198-bib-0232] Zhang, H. , Xing, L. , Rossoll, W. , Wichterle, H. , Singer, R. H. , & Bassell, G. J. (2006). Multiprotein complexes of the survival of motor neuron protein SMN with Gemins traffic to neuronal processes and growth cones of motor neurons. Journal of Neuroscience, 26, 8622–8632. 10.1523/JNEUROSCI.3967-05.2006 16914688 PMC4956918

[jnc15198-bib-0233] Zhang, K. , Donnelly, C. J. , Haeusler, A. R. , Grima, J. C. , Machamer, J. B. , Steinwald, P. , Daley, E. L. , Miller, S. J. , Cunningham, K. M. , Vidensky, S. , Gupta, S. , Thomas, M. A. , Hong, I. , Chiu, S. L. , Huganir, R. L. , Ostrow, L. W. , Matunis, M. J. , Wang, J. , Sattler, R. , … Rothstein, J. D. (2015). The C9orf72 repeat expansion disrupts nucleocytoplasmic transport. Nature, 525, 56–61. 10.1038/nature14973 26308891 PMC4800742

[jnc15198-bib-0234] Zhang, Q. , Chen, C.‐Y. , Yedavalli, V. S. R. K. , & Jeang, K.‐T. (2013). NEAT1 long noncoding RNA and paraspeckle bodies modulate HIV‐1 posttranscriptional expression. Mbio, 4, 10.1128/mBio.00596-12 PMC3560530 23362321

[jnc15198-bib-0235] Zhang, Q.‐S. , Wang, Z.‐H. , Zhang, J.‐L. , Duan, Y.‐L. , Li, G.‐F. , & Zheng, D.‐L. (2016). Beta‐asarone protects against MPTP‐induced Parkinson’s disease via regulating long non‐coding RNA MALAT1 and inhibiting α‐synuclein protein expression. Biomedicine and Pharmacotherapy, 83, 153–159. 10.1016/j.biopha.2016.06.017 27470562

[jnc15198-bib-0236] Zhang, X. , Gejman, R. , Mahta, A. , Zhong, Y. , Rice, K. A. , Zhou, Y. , Cheunsuchon, P. , Louis, D. N. , & Klibanski, A. (2010). Maternally expressed gene 3, an imprinted noncoding RNA gene, is associated with meningioma pathogenesis and progression. Cancer Research, 70, 2350–2358. 10.1158/0008-5472.CAN-09-3885 20179190 PMC2987571

[jnc15198-bib-0237] Zhang, X. , Hamblin, M. H. , & Yin, K. J. (2017). The long noncoding RNA Malat 1: Its physiological and pathophysiological functions. RNA Biology, 10.1080/15476286.2017.1358347 PMC5731810 28837398

[jnc15198-bib-0238] Zhang, X. , Xie, K. , Zhou, H. , Wu, Y. , Li, C. , Liu, Y. , Liu, Z. , Xu, Q. , Liu, S. , Xiao, D. , & Tao, Y. (2020). Role of non‐coding RNAs and RNA modifiers in cancer therapy resistance. Molecular Cancer, 19(1), 10.1186/s12943-020-01171-z PMC7050132 32122355

[jnc15198-bib-0239] Zhao, J. , Dahle, D. , Zhou, Y. , Zhang, X. , & Klibanski, A. (2005). Hypermethylation of the promoter region is associated with the loss of MEG3 gene expression in human pituitary tumors. Journal of Clinical Endocrinology and Metabolism, 90, 2179–2186. 10.1210/jc.2004-1848 15644399

[jnc15198-bib-0240] Zhu, J. , Zhang, R. , Yang, D. , Li, J. , Yan, X. , Jin, K. , Li, W. , Liu, X. , Zhao, J. , Shang, W. , & Yu, T. (2018). Knockdown of long non‐coding RNA XIST inhibited doxorubicin resistance in colorectal cancer by upregulation of miR‐124 and downregulation of SGK1. Cellular Physiology and Biochemistry, 51, 113–128. 10.1159/000495168 30439718

[jnc15198-bib-0241] Zu, T. , Gibbens, B. , Doty, N. S. , Gomes‐Pereira, M. , Huguet, A. , Stone, M. D. , Margolis, J. , Peterson, M. , Markowski, T. W. , Ingram, M. A. C. , Nan, Z. , Forster, C. , Low, W. C. , Schoser, B. , Somia, N. V. , Clark, H. B. , Schmechel, S. , Bitterman, P. B. , Gourdon, G. , … Ranum, L. P. W. (2011). Non‐ATG‐initiated translation directed by microsatellite expansions. Proceedings of the National Academy of Sciences, 108, 260–265. 10.1073/pnas.1013343108 PMC3017129 21173221

[jnc15198-bib-0242] Zu, T. , Liu, Y. , Banez‐Coronel, M. , Reid, T. , Pletnikova, O. , Lewis, J. , Miller, T. M. , Harms, M. B. , Falchook, A. E. , Subramony, S. H. , Ostrow, L. W. , Rothstein, J. D. , Troncoso, J. C. , & Ranum, L. P. W. (2013). RAN proteins and RNA foci from antisense transcripts in C9ORF72 ALS and frontotemporal dementia. Proceedings of the National Academy of Sciences, 110, E4968–E4977. 10.1073/pnas.1315438110 PMC3870665 24248382

